# DEER Spectroscopy Measurements Reveal Multiple Conformations of HIV-1 SOSIP Envelopes that Show Similarities with Envelopes on Native Virions

**DOI:** 10.1016/j.immuni.2018.06.017

**Published:** 2018-08-21

**Authors:** Beth M. Stadtmueller, Michael D. Bridges, Kim-Marie Dam, Michael T. Lerch, Kathryn E. Huey-Tubman, Wayne L. Hubbell, Pamela J. Bjorkman

**Affiliations:** 1 Division of Biology and Biological Engineering, California Institute of Technology, Pasadena, CA 91125, USA; 2 Jules Stein Eye Institute, University of California, Los Angeles, CA 90095, USA; 3 Department of Chemistry and Biochemistry, University of California, Los Angeles, CA 90095, USA

**Keywords:** HIV-1 Envelope, SOSIP, CD4, bNAbs, conformational dynamics, vaccine development, electron paramagnetic resonance, double electron-electron resonance (DEER) spectroscopy

## Abstract

HIV-1 Envelope (Env) mediates viral-host membrane fusion after binding host-receptor CD4 and coreceptor. Soluble envelopes (SOSIPs), designed to mimic prefusion conformational states of virion-bound envelopes, are proposed immunogens for eliciting neutralizing antibodies, yet only static structures are available. To evaluate conformational landscapes of ligand-free, CD4-bound, inhibitor-bound, and antibody-bound SOSIPs, we measured inter-subunit distances throughout spin-labeled SOSIPs using double electron-electron resonance (DEER) spectroscopy and compared results to soluble and virion-bound Env structures, and single-molecule fluorescence resonance energy transfer (smFRET)-derived dynamics of virion-bound Envs. Unliganded SOSIP measurements were consistent with closed, neutralizing antibody-bound structures and shielding of non-neutralizing epitopes, demonstrating homogeneity at Env apex, increased flexibility near Env base, and no evidence for the intra-subunit flexibility near Env apex suggested by smFRET. CD4 binding increased inter-subunit distances and heterogeneity, consistent with rearrangements required for coreceptor binding. Results suggest similarities between SOSIPs and virion-bound Envs and demonstrate DEER’s relevance for immunogen design.

## Highlights

• SOSIP Env apex is 3-fold symmetric and consistent with closed prefusion structures
• Unliganded Env base and CD4-bound Env apex and base exhibit flexibility
• SOSIPs retain desired properties of immunogens; e.g., burying non-neutralizing epitopes
• Results allow interpretation of smFRET studies and SOSIP and virion Env structures

## Graphical Abstract

**Figure:**
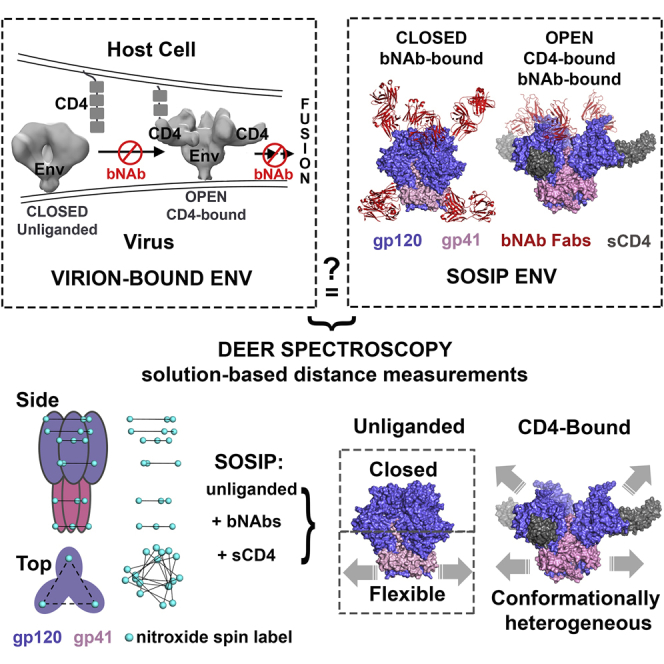

## Teaser

HIV-1 Env, the only target of neutralizing antibodies, is highly dynamic, and only snapshots of static conformations are available. Stadtmueller et al. used DEER spectroscopy to map conformations of soluble Env and its complexes with antibodies or the CD4 receptor. Results reveal similarities to virion-bound Env and buried non-neutralizing antibody epitopes, advancing knowledge of Env function and vaccine design.

## Introduction

**Figure 1:**
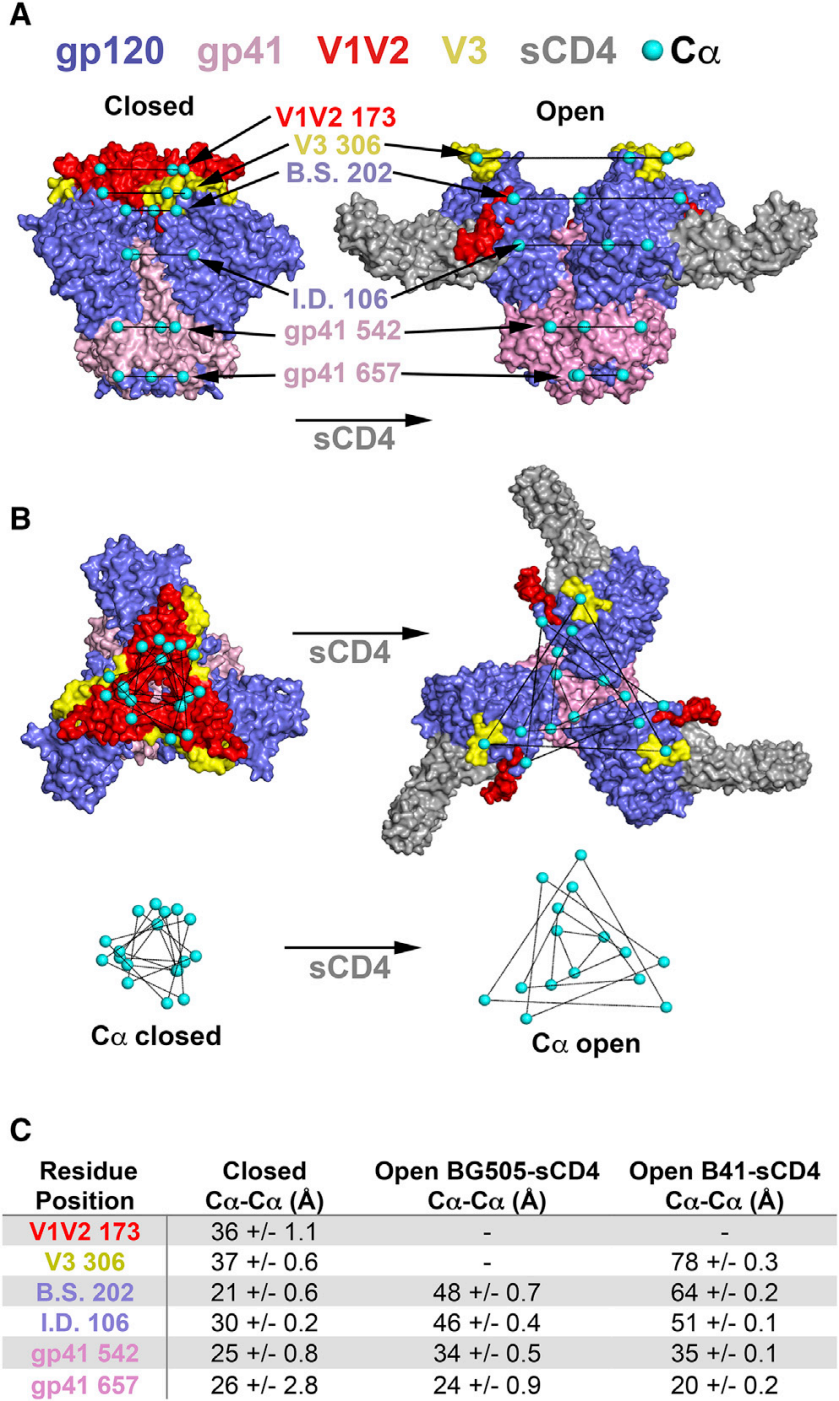
Inter-Subunit Distances between Target Site Cα Atoms (A) Side-view molecular surface representations of a closed bNAb-bound BG505 (pdb 5CEZ; PGT121 precursor and 35O22 Fabs not shown) and an open B41-sCD4 complex (pdb 5VN3; 17b Fab not shown). Spin-label site Cα atoms shown as cyan spheres. B.S., bridging sheet; I.D., inner domain. One of three copies of V1V2 and two of three bound sCD4s are visible. (B) Top view of structures shown in (A) and overlay of spin-label site Cα atoms on closed and open Env structures to illustrate changes upon sCD4 binding. (C) Table listing Env motifs, residue numbers, and measured inter-subunit distances. For closed Envs, each distance is presented as the mean and SD for measurements of SOSIP Env trimers (pdbs 5CEZ, 5T3Z, 5I8H, 5V7J, 5U7M, 5U7O) and a native (non-SOSIP) Env trimer (pdb 5FUU). For open, sCD4-bound Envs, each distance is presented as the mean and SD for the three inter-subunit distances in BG505+sCD4 (pdb 5THR) and B41+sCD4 (pdb 5VN3) structures. Dashes indicate disordered residues for which inter-subunit distances cannot be measured. See also Figure S1.

Developing a vaccine against HIV-1 requires understanding the structure and dynamics of envelope (Env) glycoproteins on virions and in soluble forms being developed as immunogens (Sanders and Moore, 2017). HIV-1 Env, a trimer of gp120-gp41 heterodimers, mediates entry into target cells by gp120 binding to the host receptor CD4, which initiates conformational changes that allow recognition of the coreceptor CCR5, resulting in gp41 rearrangements that promote fusion between the target cell and viral membranes (Ward and Wilson, 2017). Low-resolution reconstructions of Env trimers on HIV-1 virions derived by cryo-electron tomography (cryo-ET) revealed distinct Env conformations including an unliganded, closed structure in which adjacent gp120 subunits interacted to form the trimer apex and a soluble CD4 (sCD4)-bound, open conformation in which the gp120 subunits were displaced and outwardly rotated to disrupt the trimer apex (Liu et al., 2008). Subsequent crystallographic and cryo-EM structures of soluble native-like Env trimers lacking membrane and cytoplasmic domains and including stabilizing mutations (SOSIPs) (Sanders et al., 2013) in complex with broadly neutralizing antibodies (bNAbs) resulted in higher-resolution Env structures of the closed Env conformation, revealing interactions of the gp120 V1V2 motifs at the trimer apex that shield the coreceptor binding site on V3 (Ward and Wilson, 2017) (Figures 1A and 1B). Consistent with cryo-ET structures of open virion-bound Envs (Liu et al., 2008), single-particle cryo-EM structures of sCD4-bound SOSIPs demonstrated rotation and displacement of gp120s, an ∼40Å movement of V1V2 to the sides of Env trimer to reveal V3, and smaller rearrangements of gp41 (Ozorowski et al., 2017, Wang et al., 2016) (Figures 1A and 1B).

The dynamics of HIV-1 Envs on virions has been characterized by single-molecule fluorescence resonance energy transfer (smFRET) studies, in which donor and acceptor fluorophores were placed in loops within V1 and V4 of a gp120 monomer in virion-bound Env trimers, allowing for intra-subunit motions within single Env trimers to be monitored over time (Ma et al., 2018, Munro et al., 2014, Munro and Lee, 2018, Munro and Mothes, 2015). These studies suggested that virion-bound Envs transition between three primary states (States 1, 2, and 3), with State 1 (low FRET ground state with large intra-dye distances) predominating in the absence of added ligands. Transitions to State 3 (medium FRET state with intermediate intra-dye distances) were induced by the addition of sCD4 plus the coreceptor-mimicking antibody 17b after populating State 2 (high FRET state with short intra-dye distances) (Ma et al., 2018, Munro et al., 2014). Addition of bNAbs or a small molecule inhibitor of HIV-1 entry, BMS-626529 (Li et al., 2013), stabilized the low-FRET ground state, with differential effects on the intermediate- and high-FRET states (Munro et al., 2014). There is currently no correlation between atomic resolution Env structures and the smFRET-defined states, resulting in a gap in understanding the relationship between Env molecular structure and dynamics. In addition, the development of SOSIP Envs as potential immunogens (Sanders and Moore, 2017) requires assessment of whether their unliganded structures faithfully represent the conformation(s) adopted by virion-bound Envs that would be encountered in a natural infection.

To evaluate the structure and conformational flexibility of SOSIP Envs in solution, we used double electron-electron resonance (DEER) spectroscopy to probe free and liganded SOSIP Envs spin-labeled with a nitroxide free radical. DEER measures the dipolar interaction between electron spin pairs and can be analyzed to give an interspin distance distribution in the range of 17–80Å (Jeschke, 2012). The populations of distances recorded reflect molecular motion in solution, providing a snapshot of protein dynamics and conformational heterogeneity. The largest peak amplitude defines the most probable distance in a DEER distribution and provides information on the dominant structural state in the population, while multiple peaks indicate conformational heterogeneity and peak widths are related to the internal flexibility of each conformation and the attached spin label (Hubbell et al., 2000, Hubbell et al., 2013). The accuracy of peak distance and width decrease as a function of interspin distance; 17–65Å distances can be assigned with confidence, whereas distances > 65Å are detected with less accuracy (e.g., +/−10Å) (Jeschke, 2012).

Here we report DEER-derived inter-subunit distances and heterogeneity in unliganded SOSIP Envs and their complexes with sCD4, bNAbs, and a small molecule inhibitor. We also report an intra-subunit measurement between spin labels at positions similar to where dyes were introduced in smFRET studies of virion-bound Envs (Ma et al., 2018, Munro et al., 2014). These results inform models of Env dynamics relevant to developing SOSIP immunogens and to understanding conformational changes in Env-mediated membrane fusion.

## Results

We chose to investigate two well-characterized SOSIP trimers, the clade A BG505 (Sanders et al., 2013) and clade B B41 (Pugach et al., 2015), because these SOSIP Envs have been characterized structurally in closed and sCD4-bound open states (Ozorowski et al., 2017, Wang et al., 2016) and both are being developed as immunogens (Sanders et al., 2015). For each DEER experiment we used site-directed spin labeling (Hubbell et al., 2013) to introduce a single cysteine into a gp120-gp41 protomer of the BG505 or B41 SOSIPs and then covalently attached a nitroxide spin label bearing the “V1” side chain (Khramtsov et al., 1989) (indicated as an asterisk,^∗^) (Figure S1A). Once attached, the V1 spin label is about the size of an amino acid side chain and contributes limited width to DEER distance distributions (Toledo Warshaviak et al., 2013) (Figures S1A and S1B). This approach results in the attachment of three spin labels per trimer, which form the vertices of an equilateral triangle if located in a symmetric portion of Env (Figure 1B), or the vertices of an isosceles or scalene triangle if located in a region that lacks 3-fold symmetry and/or adopts multiple conformations. Accordingly, DEER measurements were expected to result in one distance corresponding to the sides of an equilateral triangle or ≥ 2 distances corresponding to the sides of an asymmetric triangle or equilateral triangles of different sizes.

We selected spin labeling sites from solvent-exposed residues in defined secondary structures (β strand or α-helix; not loops) in the following Env structural motifs in the apex or base regions: (1) (apex) gp120 V1V2, which mediates inter-subunit contacts that form the apex of closed Env trimers and repositions upon Env binding to sCD4 (Wang et al., 2016); (2) (apex) gp120 V3, which is shielded by V1V2 in the closed Env conformation and is exposed to provide coreceptor-binding sites upon Env binding to sCD4 (Ward and Wilson, 2017); (3) (apex) gp120 bridging sheet at the base of V1V2, which undergoes structural rearrangements upon CD4 binding that stabilize open conformations (Ozorowski et al., 2017, Wang et al., 2016); (4) (base) gp120 inner domain, which interacts with gp41 (Ward and Wilson, 2017); and (5) (base) gp41, portions of which undergo conformational changes upon binding to sCD4 (Ozorowski et al., 2017) (Figures 1A and 1B).

To evaluate the positions of spin labeling sites among published structures, we measured inter-subunit Cα distances from crystal and cryo-EM structure coordinates and determined a mean distance and SD for each site (Figure 1C). Measurements included seven closed Envs (six SOSIPs) (Ward and Wilson, 2017) and one native (non-SOSIP) Env (Lee et al., 2016), each complexed with one or more bNAb Fabs, B41 SOSIP bound to the CD4 binding site (CD4bs) bNAb b12 (B41-b12 complex) (Ozorowski et al., 2017), BG505 SOSIP bound to sCD4, the CD4-induced (CD4i) coreceptor-mimicking antibody 17b, and the gp120-gp41 interface bNAb 8ANC195 (BG505-sCD4-17b-8ANC195 complex) (Wang et al., 2016), and B41 SOSIP bound to sCD4 and 17b (B41-sCD4-17b complex) (Ozorowski et al., 2017). For closed Envs structures, the mean inter-subunit distance SD were ∼1Å or less, with the exception of residue 657 in gp41, whose position was different in two structures (pdb codes 5U70 and 5U7M), resulting in a 2.8Å SD (Figure 1C). We used these coordinate-derived measurements as references to compare with DEER-derived distances, noting that a coordinate-derived Cα-Cα measurement and an experimental DEER distance may differ by several Ångstrom yet represent the same structure because V1 side chain rotamers could contribute to the DEER distance (Figure S1B).

### V1V2 Measurements Correspond to Closed and Open Env Structures

**Figure 2:**
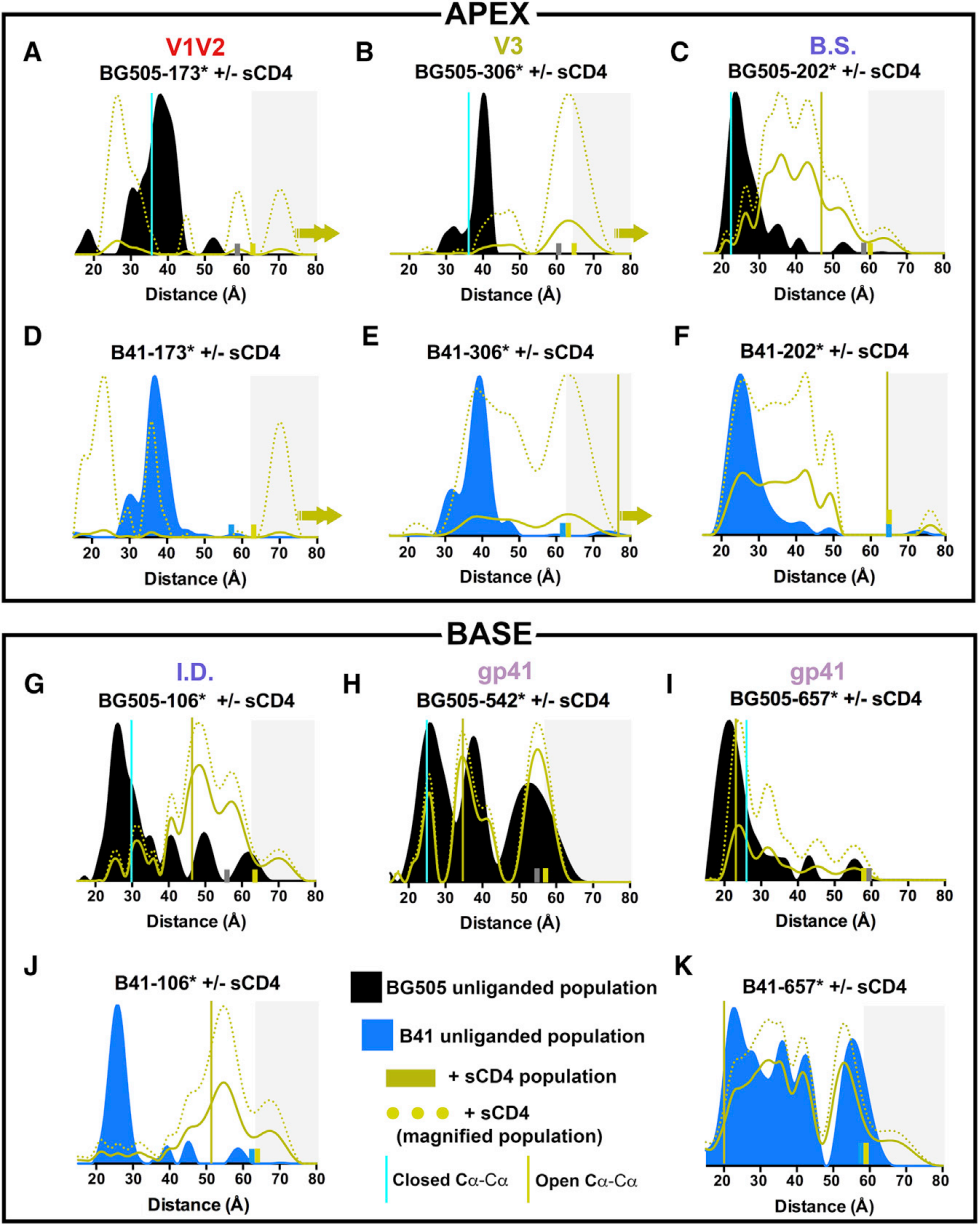
DEER Detects Conformational Changes between Unliganded and sCD4-Bound Envs (A–F) Distance distributions for spin labels at Env apex in unliganded and sCD4-bound BG505 and B41 Envs. (G–K) Distributions for spin labels at Env base in unliganded and sCD4-bound BG505 and B41 Envs. The heights of solid colored distributions (BG505 unliganded, B41 unliganded, +sCD4) were normalized for total distribution area scaled by the depth of modulation (DOM) (Figure S2) to reflect the fraction of total spin pairs within the DEER detection limit, and thus, monitor changes in the detectable populations at each distance. For sCD4-bound samples, the heights of dotted distributions were scaled to the amplitude of the unliganded sample, providing a magnified population to facilitate visualization of peak distances and FWHMs. Vertical lines indicate the mean inter-subunit distance (Figure 1) for each site in structures of closed Env (cyan lines) and in open sCD4-bound Envs (yellow lines). Small bars (gray, unliganded BG505; blue, unliganded B41; yellow, Env plus sCD4) indicate the limit of reliable distance measurements for each dataset; populations not reliably determined are indicated by a gray background. Upon the addition of sCD4, the number of spin pairs in the detection range decreased sharply in some samples (highlighted by arrows in liganded populations) as spins moved beyond the detection range; the distance distribution shown is only for the populations within the detection range. For BG505-173^∗^–sCD4 and B41-173^∗^–sCD4, two arrows indicate that ∼90% of the spins moved out of range to longer distances. See also Figure S2.

To evaluate Env flexibility and heterogeneity in V1V2, which forms the closed trimer apex and is repositioned upon CD4 binding, we spin-labeled BG505 β strand residue 173 to make BG505-173^∗^. Distance distributions calculated from DEER spectra for unliganded BG505-173^∗^ revealed a most probable distance peak at 38Å, with a full width at half maximum (FWHM) of ∼10Å broadening to ≥ 15Å at its base (Figures 2A and S2), consistent with Cα-Cα measurements of closed Env structures (mean = 36Å) (Figure 1C) and suggesting 3-fold symmetry and detectable, but limited, structural heterogeneity. Unliganded B41-173^∗^ also exhibited a most probable distance at 38Å, similar to the BG505-173^∗^ peak, albeit with a reduced FWHM and a lower probability shoulder in the 29–35Å range, indicative of structural homogeneity in V1V2 (Figures 2D and S2).

The addition of sCD4 to BG505-173^∗^ and B41-173^∗^ induced a large reduction of interspin pairs, suggesting that the majority of 173^∗^ spin labels moved beyond the ∼80Å limit of DEER detection, consistent with cryo-EM structures of sCD4-bound SOSIP Envs in which V1V2 is displaced to the sides of Env trimer and in which the three copies of residue 173 are separated by ∼110Å) (Ozorowski et al., 2017; H. Wang and P.J.B., unpublished data; Wang et al., 2016) (Figures 2A and 2D; S2). We also detected a small heterogeneous population (< 10% of total) of short (∼25Å) and long (> 65Å) interspin distance peaks for both samples (Figures 2A and 2D), which may reflect defined structures in a sCD4-bound conformation.

### The V3 Region Is Rigid When Closed and Flexible When CD4-Bound

We investigated the conformational flexibility of V3, the binding site for coreceptor, which is buried beneath V1V2 in closed structures and exposed upon CD4 binding (Ward and Wilson, 2017), by spin labeling BG505 residue 306 (Figure 1). DEER distributions for unliganded BG505-306^∗^ exhibited a narrow peak at 40Å with a < 5Å FWHM (Figure 2B), indicating that the unliganded BG505 Env structure is 3-fold symmetric and rigid with respect to V3. Upon binding to sCD4, a decrease in interspin signal indicated that some spin labels moved out of range, while a smaller population exhibited a peak at 65Å (Figure 2B). B41-306^∗^ and B41-306^∗^–sCD4 distance distributions yielded similar results, with a narrow distribution at 38Å for unliganded samples and increased heterogeneity characterized by a broad peak centered at 65Å upon the addition of sCD4 (Figure 2E). These results are consistent with closed and sCD4-bound Env structures: the inter-subunit distance separating residue 306 Cα atoms in closed bNAb-bound Env (Pancera et al., 2014) is 37Å, and residue 306 is either disordered (BG505-sCD4-17b-8ANC195) or separated by 78Å (B41-sCD4-17b) in sCD4-bound Env structures (Ozorowski et al., 2017, Wang et al., 2016) (Figure 1C). The ∼13Å difference in inter-subunit distances between B41-306^∗^-sCD4 DEER measurements (65Å) and the B41-sCD4-17b cryo-EM structure (78Å) suggests that the B41-sCD4 complex can adopt shorter inter-subunit distances between V3 loops in the absence of 17b Fab, which may stabilize a larger inter-subunit separation though interactions near residue 306.

### Conformational Heterogeneity in the gp120 Bridging Sheet Increases upon CD4 Binding

We next labeled residue 202 in the gp120 β3 strand that becomes part of the bridging sheet upon sCD4 binding (Ozorowski et al., 2017, Wang et al., 2016). Distance distributions for unliganded BG505-202^∗^ revealed a most probable distance at 24Å with a ∼10Å FWHM (Figure 2C), consistent with closed bNAb-bound Env structures (Figure 1C). Adding sCD4 to BG505-202^∗^ broadened the FWHM to 20Å with an overall 50Å span; it also shifted most probable distances, resulting in peaks at 36Å and 42Å (Figure 2C). The B41-202^∗^ and BG505-202^∗^ distance distributions were essentially superimposable, and the B41-202^∗^–sCD4 distance distribution also showed that addition of sCD4 increased conformational heterogeneity, including a new peak at 44Å (Figure 2F). The emergence of long distance peaks upon sCD4 binding is consistent with residue 202 Cα atoms being separated by 48Å (BG505-sCD4-17b-8ANC195) or 64Å (B41-sCD4-17b) in sCD4-bound Env structures (Ozorowski et al., 2017, Wang et al., 2016) (Figure 1C), and the existence of multiple distances suggests the existence of sCD4-bound conformations that have not been observed in X-ray and cryo-EM Env structures.

### The gp120 Inner Domain Exhibits Conformational Heterogeneity

To study the gp120 inner domain, a region near the middle of Env trimer that contacts neighboring gp120 subunits and the gp41 subunit in the same protomer (Ward and Wilson, 2017), we spin-labeled gp120 residue 106. Unliganded BG505-106^∗^ distance distributions exhibited a 27Å most probable distance (Figure 2G), consistent with Cα-Cα distances measured in bNAb-bound BG505 crystal structures (30Å) (Figure 1C). We also observed a population of lower probability (∼25%) peaks at 35Å, 40Å, 50Å, and 61Å, indicating conformational heterogeneity in the inner domain of unliganded SOSIP Env (Figure 2G). The addition of sCD4 induced a distance distribution shift in which peaks at 40Å, 48Å, and 58Å were populated, with the 48Å peak being the most probable. These results are consistent with residue 106 Cα atoms being separated by 46Å (BG505-sCD4-17b-8ANC195) or 51Å (B41-sCD4-17b) in sCD4-bound Env structures (Ozorowski et al., 2017, Wang et al., 2016) (Figure 1C) and also suggest the presence of sCD4-bound conformations in the inner domain of unliganded Env (Figure 2G). B41-106^∗^ exhibited a similar distance distribution to its BG505-106^∗^ counterparts when unliganded and sCD4-bound (Figure 2J). However, unliganded B41-106^∗^ appeared more homogeneous, with a larger population of the dominant peak corresponding to closed Env structures.

### The gp41 Base Exhibits Conformational Flexibility

We also spin-labeled gp41 residues 542 and 657 toward or at the base of the SOSIP trimer (Figure 1C). Unliganded BG505-542^∗^ exhibited a broad multimodal distance distribution (Figure 2H), with the shortest distance peak (26Å) consistent with closed Env structures (Figure 1C). The addition of sCD4 had relatively little effect on the overall heterogeneity of BG505-542^∗^ distribution (Figure 2H). Notably, the most probable peak at ∼34Å that appeared in both liganded and sCD4-bound BG505-542^∗^ is consistent with distance measurements in sCD4-bound Env structures (Ozorowski et al., 2017, Wang et al., 2016) in which residue 542 Cα atoms are separated by 34Å (BG505-sCD4-17b-8ANC195) or 35Å (B41-sCD4-17b) (Figure 1C), suggesting the presence of sCD4-bound conformation(s) in unliganded gp41.

Distance distributions for unliganded BG505-657^∗^ showed a narrow peak at 22Å with a shoulder at 27Å and a ∼10Å FWHM (Figure 2I), consistent with the 26Å measured distance in closed Env structures (Figure 1C). BG505-657^∗^ incubated with sCD4 exhibited a similar distribution, characterized by peaks at 25Å and 32Å (Figure 2I). In contrast, B41-657^∗^ exhibited a broad multimodal distribution with multiple peaks ranging from 20–65Å, and the addition of sCD4 resulted in few changes other than minor differences in relative peak heights (Figure 2K). While the unliganded BG505-657^∗^ data were consistent with bNAb-bound closed trimer structures (26 ± 2.8Å mean inter-subunit distance separating residue 657 Cα atoms), the BG505-657^∗^–sCD4, B41-657^∗^, and B41-657^∗^–sCD4 data exhibited heterogeneity characterized by distances longer than those measured in CD4-bound Env structures (Figure 1C; 2I and 2K).

### bNAbs and Inhibitors Have Differential Effects on SOSIP Env Conformations

**Figure 3:**
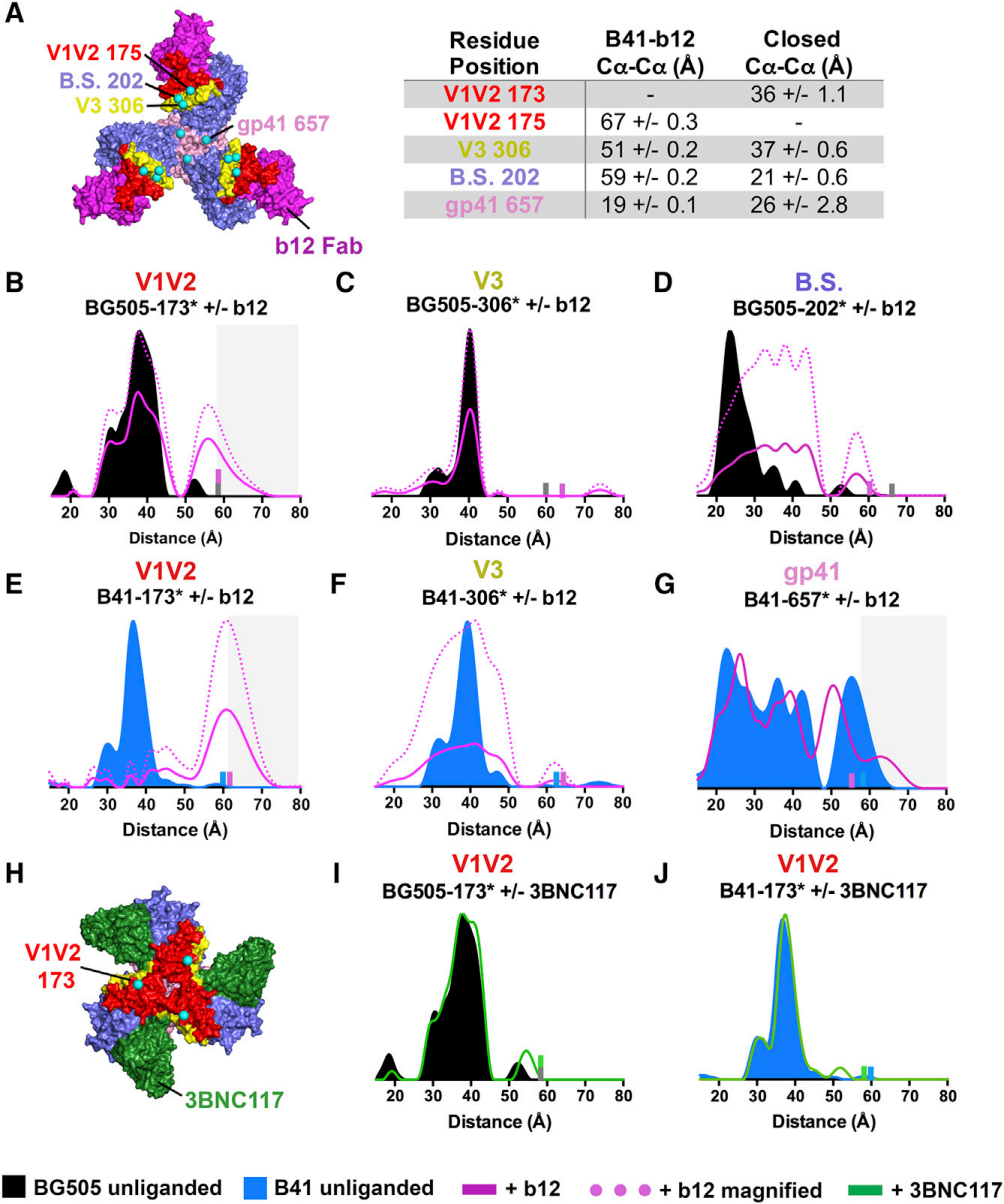
CD4bs bNAbs Induce Distinct Structural Changes (A) Left shows structure of a B41-b12 complex (pdb 5VN8) with b12 V_H_-V_L_ in magenta and the positions of spin-labeled sites overlaid as cyan spheres. Right shows measured inter-subunit distances separating the indicated residues in b12-bound and closed structures. Each distance is presented as the mean and SD from multiple measurements as described in the Figure 1C legend. (B–G) Distance distributions for labeled BG505 (B–D) and labeled B41 (E–G) Envs in the presence and absence of b12. (H) Structure of a BG505-3BNC117 complex (pdb 5V8M) with 3BNC117 V_H_-V_L_ shown in green and the positions of spin-labeled sites overlaid as cyan spheres. (I and J) Distance distributions for unliganded and 3BNC117-bound Envs for BG505-173^∗^ (I) and B41-173^∗^ (J). Small bars (gray, BG505 unliganded; blue, B41 unliganded; magenta, Env plus b12; green, Env plus 3BC117) indicate the limit of reliable distance measurements for each dataset; populations not reliably determined are indicated by a gray background. Distributions were normalized and shown as described in the Figure 2 legend. See also Figure S3.

**Figure 4:**
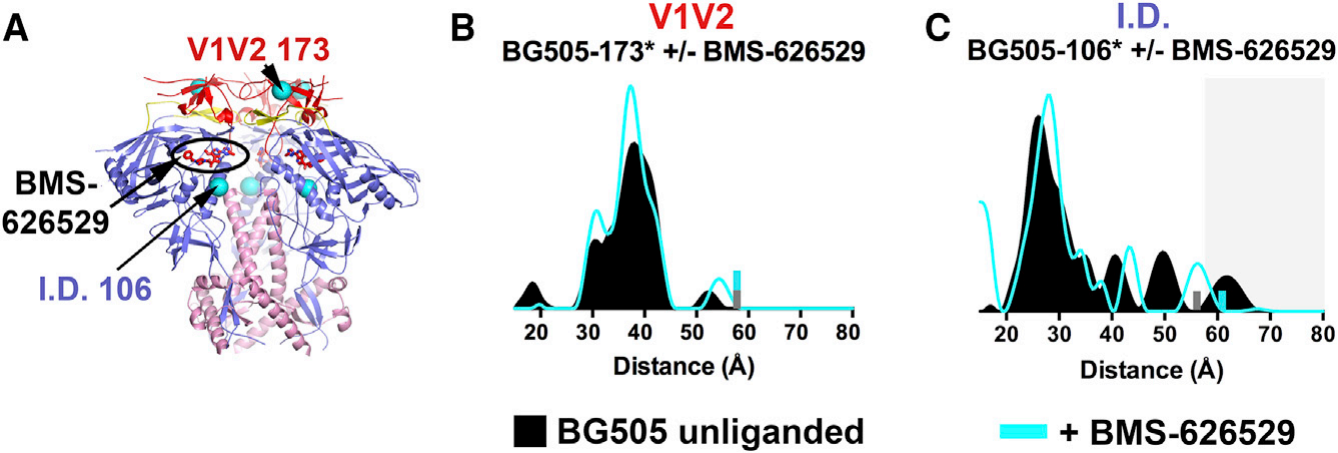
Small Molecule Inhibitor BMS-626529 (A) BG505–BMS-626529 structure (pdb 5U7O; PGT122 and 35O22 Fabs not shown) showing the BMS-626529 binding sites (one site indicated) with the positions of spin-labeled sites overlaid as cyan spheres. (B and C) Distance distributions for BG505-173^∗^ (B) and BG505-106^∗^ (C) in the presence and absence of BMS-626529. Small bars (gray, BG505 unliganded; cyan, Env plus BMS-626529) indicate the limit of reliable distance measurements for each dataset; populations not reliably determined are indicated by a gray background. Distance distributions were normalized as described in Figure 2 legend. See also Figure S4.

**Figure 5:**
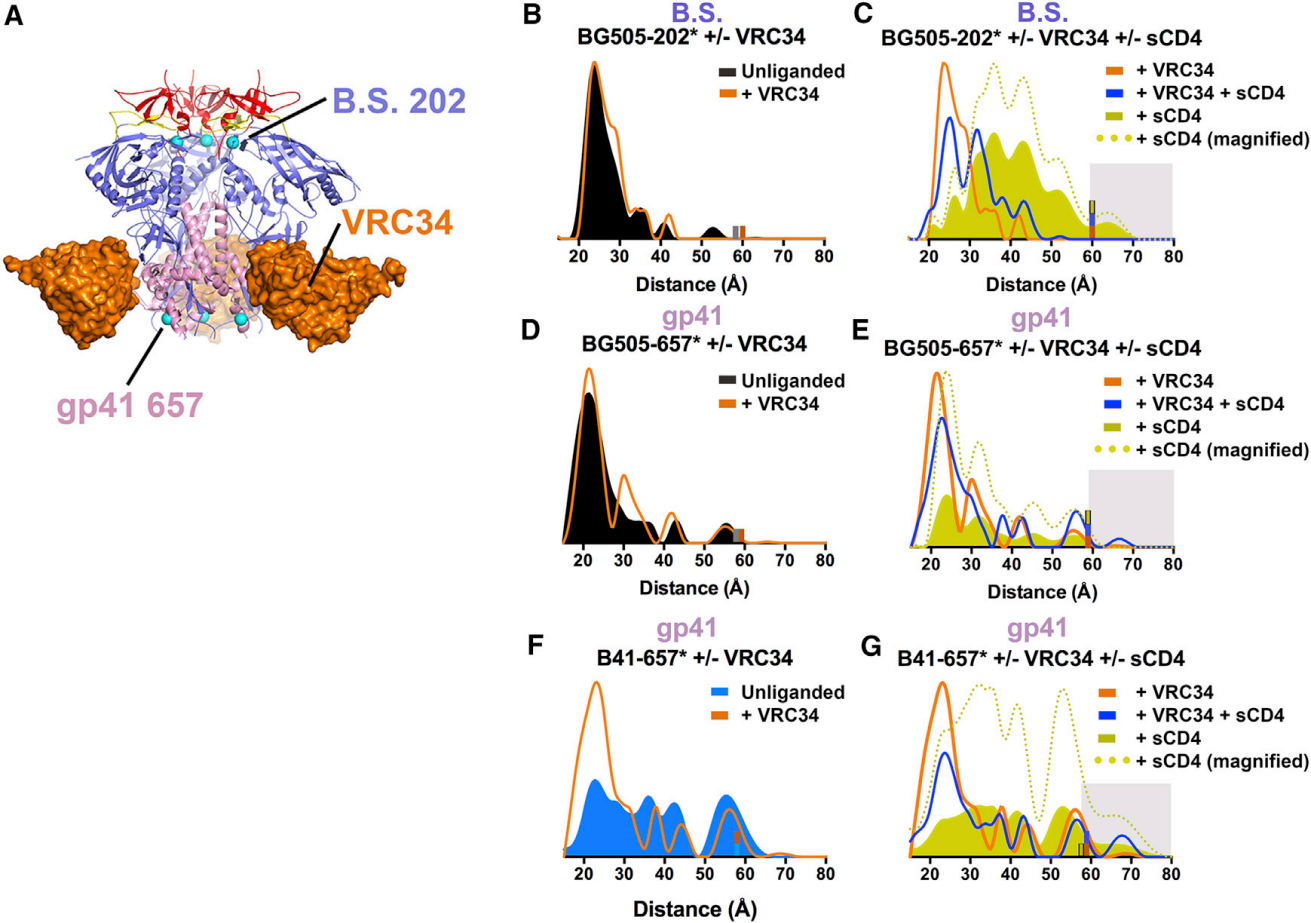
Effects of bNAb VRC34 (A) BG505-VRC34 crystal structure (pdb 5I8H; PGT122 Fab not shown) with spin-labeled sites overlaid as cyan spheres. (B–G) Distance distributions for BG505-202^∗^ (B and C) and BG505-657^∗^ (D and E) and B41-657^∗^ (F and G). (B), (D), and (F) show distance distributions for labeled BG505 or B41 in the presence and absence of VRC34. (C), (E), and (F) show distance distributions for labeled Env-VRC34 complexes in the presence and absence of sCD4 and comparisons to distributions for labeled Env-sCD4 complexes from Figure 2. Small bars (gray, BG505 unliganded; blue, B41 unliganded; orange, Env plus VRC34; blue, Env plus VRC34 and sCD4; yellow, Env plus sCD4) indicate the limit of reliable distance measurements for each dataset; populations not reliably determined are indicated by a gray background. Distance distributions for BG505-sCD4 are shown for comparison. Distance distributions were normalized as described in the Figure 2 legend and magnified distributions (dotted line; scaled to the amplitude of the unliganded sample) are shown for sCD4 only-containing samples to facilitate comparison of peak distances among samples. See also Figure S5.

To investigate how bNAb and small molecule ligands influence Env conformations, we collected DEER spectra for spin-labeled Envs following incubation with the CD4-binding site (CD4bs) bNAbs b12 (Burton et al., 1991) or 3BNC117 (Scheid et al., 2011), the small molecule HIV-1 entry inhibitor BMS-626529 (Li et al., 2013), and the gp41 fusion peptide-binding bNAb N123-VRC34.01 (Kong et al., 2016) (hereafter VRC34) (Figures 3, 4, and 5; S3–S5).

b12 binding to virion-bound and SOSIP Envs stabilizes an open conformation of gp120 subunits and rearrangements in gp41 (Liu et al., 2008, Ozorowski et al., 2017), similar to changes observed upon sCD4 binding, although the ∼40Å V1V2 movement and rearrangement of the gp120 bridging sheet that results from CD4 binding (Wang et al., 2016) does not occur in b12-bound Env (Ozorowski et al., 2017) (Figure 3A). By contrast, binding of VRC01-class CD4bs bNAbs such as 3BNC117 does not alter closed SOSIP structures (Lee et al., 2017, Stewart-Jones et al., 2016). We collected DEER data for b12 complexes with BG505-173^∗^, B41-173^∗^, BG505-202^∗^, BG505-306^∗^, B41-306^∗^, and B41-657^∗^, and 3BNC117 complexes with BG505-173^∗^ and B41-173^∗^ (Figure 3; S3).

Binding of b12 altered distance distributions associated with most sites. The BG505-173^∗^–b12 and B41-173^∗^–b12 DEER distributions showed new peaks at 55Å and 62Å, respectively (Figures 3B and 3E), consistent with the B41-b12 cryo-EM structure in which the Cα-Cα distance between the three copies of residue 175 is 67Å (173 is disordered in this structure) (Figure 3A). The addition of b12 had minimal effects on BG505-306^∗^, but increased the heterogeneity of B41-306^∗^, as evidenced by the broader distribution surrounding the 38Å peak that was observed in unliganded B41-306^∗^ (25Å FWHM for b12-bound B41-306^∗^; 10Å FWHM for unliganded B41-306^∗^) (Figures 3C and 3F). This result suggests that upon b12 binding, B41 residue 306 can adopt conformations other than that observed in the cryo-EM structure in which the Cα-Cα separation distance is 51Å. For BG505-202^∗^, b12 binding resulted in a multimodal distribution with a range of longer-distance peaks spanning ∼31–57Å (Figure 3D). Similar to unliganded B41-657^∗^, the B41-657^∗^–b12 DEER data exhibited a multimodal distribution spanning ∼30Å; however, the B41-657^∗^–b12 most probable distances were shifted from their unliganded counterparts, suggesting that b12 binding to V1V2 resulted in rearrangements at the base of Env (Figure 3G).

By contrast with the b12 results, incubation of BG505-173^∗^ and B41-173^∗^ with 3BNC117 Fab revealed distance distributions indistinguishable from their unliganded counterparts, indicating that 3BNC117 binding did not alter the conformation of V1V2 (Figures 3I and 3J), consistent with a BG505 SOSIP-3BNC117 structure (Lee et al., 2017) (Figure 3H).

BMS-626529 is a small molecule HIV entry inhibitor (Li et al., 2013) that prevents CD4 binding by interacting with gp120 through an induced pocket involving bridging sheet residues located near the inner domain (Pancera et al., 2017) (Figure 4A). The crystal structure of a bNAb-bound BG505–BMS-626529 complex (Pancera et al., 2017) (Figure 4A), although superimposable with other bNAb-BG505 complexes except for differences in the C-terminal helix near gp41 residue 657, showed BMS-626529 binding gp120 near an inner domain helix that contains gp120 residue 106. Consistent with the crystal structure, addition of BMS-626529 did not markedly influence the distance distribution of BG505-173^∗^, although the FWHM narrowed by ∼5Å (Figures 4B and S4) suggesting reduced heterogeneity. BMS-626529 also altered the unliganded BG505-106^∗^ distance distribution in a manner suggesting reduced heterogeneity, in this case changing both long and short distance peaks (Figure 4C).

To explore how the fusion peptide-binding bNAb VRC34 (Kong et al., 2016) (Figure 5A) affects Env conformations, we collected DEER data for BG505-202^∗^–VRC34 and BG505-657^∗^–VRC34 complexes in the presence and absence of sCD4 (Figure 5, S5). The distance distribution of BG505-202^∗^ incubated with VRC34 Fab was similar to unliganded BG505-202^∗^, both exhibiting a 22Å most probable distance (Figure 5B). For the BG505-202^∗^–VRC34–sCD4 complex, the distance distribution was broader than the distribution for unliganded BG505-202^∗^ (FWHM ∼15Å compared to ∼10Å for unliganded), yet narrower than the B505-202^∗^–sCD4 distributions and characterized by a new peak at 32Å (Figure 5C). These results suggest that VRC34 inhibited some, but not all, sCD4-induced movements in the gp120 bridging sheet, and that BG505-SOSIP-VRC34-sCD4 complexes adopt conformations that have not been observed in X-ray or cryo-EM structures.

In contrast to the similarities between the BG505-202^∗^ and BG505-202^∗^–VRC34 distance distributions (Figure 5B), the BG505-657^∗^–VRC34 distance distribution showed differences from unliganded BG505-657^∗^ even in the absence of sCD4, exhibiting a second most probable distance at 30Å in addition to the 22Å most probable distance in both VRC34-bound and unliganded BG505-657^∗^ (Figure 5D). The BG505-657^∗^–VRC34–sCD4 distance distribution showed only minor differences compared to BG505-657^∗^–VRC34, suggesting few effects in gp41 following sCD4 addition to an Env-VRC34 complex (Figure 5E). The addition of VRC34 to B41-657^∗^, which exhibited a broad multimodal distribution when unliganded (Figure 2K), increased the population of a short-distance peak at 22Å (Figure 5F). The B41-657^∗^–VRC34–sCD4 distance distribution showed only minor differences compared to B41-657^∗^–VRC34, but was distinct from B41-657^∗^ + sCD4 (Figure 5G). Taken together, these results are consistent with VRC34 inhibiting CD4-induced conformational changes that expose the coreceptor binding site (Kong et al., 2016) and suggest that VRC34 can limit mobility in B41 gp41.

### Intra-Subunit Flexibility between SOSIP V1V2 and V4 Is Limited and Contrasts with smFRET Results

**Figure 6:**
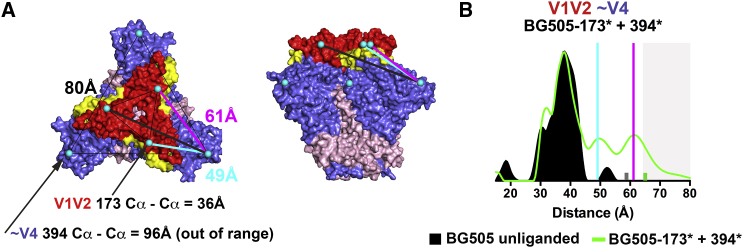
Intra-Protamer V1-V4 Distance Measurements Reveal One Distance (A) Closed BG505 structure (pdb 5CEZ) colored as in Figure 1 with spin-labeled sites (three 173^∗^ labels in V1V2 and three 394^∗^ labels in the β strand preceding V4) overlaid as cyan spheres. A single set of possible 173^∗^–394^∗^ distances are drawn as colored lines between a residue 394 Cα atom in one protomer and the three residue 173 Cα atoms in the trimer. The analogous distances measured from the other two residue 394 Cα atoms are equivalent in closed Env structures. Distances shown on the figure are the mean of measurements from the seven closed Env structures used for inter-subunit measurements in Figure 1C. SD for these measurements were under 1Å. (B) DEER distance distributions for unliganded BG505-173^∗^ and unliganded BG505-173^∗^+394^∗^. The positions of the 49Å and the 61Å distances shown in (A) are shown as cyan and magenta lines, respectively on the distance distribution. Measurements on BG505-394^∗^ did not produce detectable signal (Figure S6), indicating that spin labels were out of DEER range. Small bars (gray, BG505-173^∗^ unliganded; green, BG505-173^∗^ + 394^∗^ unliganded) indicate the limit of reliable distance measurements for each dataset; populations not reliably determined are indicated by a gray background. BG505-173^∗^ and BG505-173^∗^ + 394^∗^ distributions were scaled to the same amplitude because they were independent protein variants. See also Figure S6.

To characterize conformational states within a single Env subunit and compare with smFRET studies of virion-bound Envs (Ma et al., 2018, Munro et al., 2014), we produced BG505 variants in which each gp120 included two spin-labeled sites: 173^∗^ (in V1V2) and 394^∗^ (in β18 strand immediately upstream of V4) (Figure 6A). This resulted in Env trimers with six spin labels, which include inter-subunit distances between the three copies of each spin label, and both inter- and intra- subunit distances between 173^∗^ and 394^∗^ (Figures 6A and S6). Based on measurements between Cα atoms in closed Env structures (Ward and Wilson, 2017), we expected five possible distances, two of which (394^∗^ inter-subunit distances and one of the 173^∗^–394^∗^ inter-subunit distances) should be > 80Å and thus undetectable in DEER experiments) (Figure 6A). Indeed, there was no detectable DEER signal in BG505-394^∗^ (Figure S6), and distance distributions from BG505-173^∗^+394^∗^ variants exhibited three peaks, one at 38Å, which overlapped with the BG505-173^∗^ distributions, a peak at 50Å, corresponding to the measured 173^∗^-394^∗^ intra-subunit distance (mean = 49Å), and a peak at 62Å, corresponding to the 173^∗^-394^∗^ inter-subunit distance (mean = 61Å) (Figures 6B and S6). The close match between the BG505-173^∗^+394^∗^ distance distribution and SOSIP Env structures (Figures 6A and 6B), combined with the lack of additional peaks that would indicate asymmetry and/or additional conformations, suggest that BG505 SOSIP exists in a single, symmetric conformation with respect to distances between the V1V2 and V4 regions.

## Discussion

HIV-1 SOSIP Envs (Sanders et al., 2013), are being evaluated as potential immunogens to elicit anti-HIV-1 bNAbs (Escolano et al., 2016, Medina-Ramírez et al., 2017, Steichen et al., 2016). SOSIPs were designed to mimic the closed, prefusion state of virion-bound HIV-1 Envs in order to induce relevant immune responses in humans (Sanders and Moore, 2017), thus understanding their structures compared with virion Env trimers and evaluating their conformation(s) is critical for vaccine design, interpreting results from clinical trials, and understanding dynamics related to Env functions in receptor recognition and membrane fusion.

**Figure 7:**
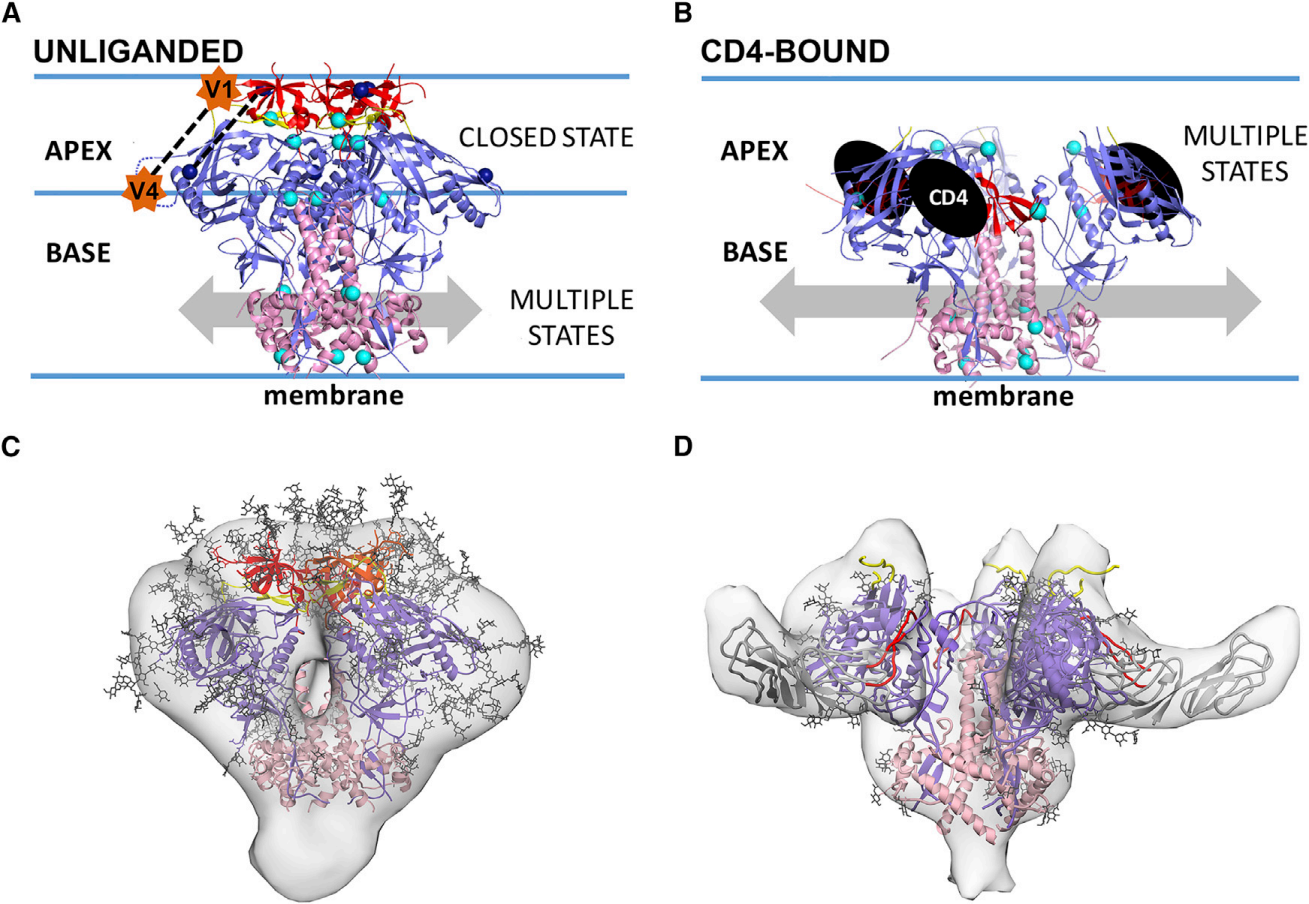
Model for Env Conformational Dynamics (A) Unliganded SOSIP Envs are characterized by a closed, three-fold symmetric and homogeneous apex conformation and heterogeneous base conformations. Spin label locations for inter-subunit DEER experiments are shown as cyan spheres; spin labels used to measure an intra-subunit distance (V1V2 173^∗^ to ∼V4 394^∗^; Figure 6) are navy blue and connected by a dotted line. The approximate locations of residues 136 (in V1) and 400 (V4; disordered in SOSIP structures) where peptide linkers were inserted to attach smFRET dyes that evaluated intra-subunit dynamics (Munro et al., 2014) are shown as orange stars connected by a dotted line. (B) The sCD4-bound SOSIP is characterized by open heterogeneous conformations at the apex and the base. (C) Comparison of closed SOSIP Env structure (cartoon representation with N-glycans depicted as sticks (pdb 5T3X) and EM density map of virion-bound unliganded Env (EMDB 5019) derived by cryo-ET/subtomogram averaging. (D) Comparison of open B41 SOSIP complexed with sCD4 and 17b (pdb 5VN3) and EM density map of open virion-bound Env complexed with sCD4 plus 17b (EMDB 5020). Coordinates and EM density for 17b Fabs were omitted for clarity. The central cavities in the virion-bound EM density maps in (C) and (D) are artifacts of the low resolution (∼20Å) of the cryo-ET structures (Bartesaghi et al., 2013).

Here we investigated SOSIP conformations in solution using DEER spectroscopy, comparing results to static crystal and cryo-EM structures of SOSIP and non-SOSIP Env trimers (Ward and Wilson, 2017), low resolution cryo-ET structures of virion-bound Envs (Liu et al., 2008), and smFRET studies of Env dynamics on viruses (Ma et al., 2018, Munro et al., 2014) (Figure 7). We note that within the ∼20Å resolution limitation of the cryo-ET structures (Liu et al., 2008), the distinct closed and open conformations of Envs on HIV-1 virions superimpose with structures of analogous SOSIP Env conformations (Harris et al., 2011). DEER distance distributions were consistent with coordinate measurements of closed (bNAb-bound) and open sCD4-bound SOSIP Env structures demonstrating that (1) unliganded SOSIP Envs resemble closed bNAb-bound SOSIP structures, (2) SOSIPs can adopt relevant receptor-bound conformations, and (3) DEER measurements can be used as reporters of structural changes and conformational landscapes in SOSIP Envs. Given the similarity of SOSIP and virion-bound Env structures, the DEER results can be extended to allow speculation about viral Env dynamics that are relevant to recognition by antibodies and Env’s function in fusion between the viral and host membranes.

DEER measurements of unliganded BG505 and B41 SOSIPs at the trimer apex (V1V2 173^∗^, bridging sheet 202^∗^, and V3 306^∗^) were consistent with bNAb-bound SOSIP structures to within a few Ångstroms, confirming that cryo-EM and X-ray structures faithfully report the dominant solution conformation of SOSIP Envs at the trimer apex. The DEER distributions were relatively narrow, suggesting minimal conformational heterogeneity and/or flexibility in the trimer apex. Indeed, the remarkably narrow BG505-306^∗^ distribution indicated a highly rigid structure and tight 3-fold symmetry. On virions, 3-fold symmetry at a closed Env trimer apex is likely important to prevent exposing the coreceptor binding site on V3 prior to engagement with a host CD4 receptor (Ward and Wilson, 2017); thus SOSIPs reproduce this presumed functional property of virion Envs. In addition, these results demonstrate the ability of DEER to detect potential V3 region exposure, thereby providing a means to guide design of immunogens that do not expose non-neutralizing antibody epitopes.

DEER measurements at sites distal from the trimer apex (gp120 inner domain 106^∗^, gp41 542^∗^, and gp41 657^∗^) were also consistent with bNAb-bound SOSIP structures, but the distributions were broader and multimodal compared with the narrow distributions of apex measurements. Thus, motifs at the Env base are conformationally heterogeneous and sample conformations that are not apparent in closed structures. Heterogeneity was pronounced for B41-657^∗^, suggesting more flexibility in B41’s trimer base than the base of BG505, consistent with reports that B41 SOSIP is more flexible than BG505 (Pugach et al., 2015). However, our apex measurements indicated that B41 flexibility excluded its trimer apex.

Conformational heterogeneity associated with measurements distal from the apex could arise from local flexibility and/or deviations from 3-fold symmetry. Notably, structurally-uncharacterized distances overlapped with distances that were populated upon the addition of sCD4, suggesting that regions of the gp120 inner domain and gp41 transiently sample sCD4-bound conformations when unliganded, but that conformational heterogeneity is not transferred to the apex in the absence of sCD4. If this property of SOSIP Envs extends to virion Envs, decoupling between the Env base and apex might prevent membrane perturbations from triggering apex opening until CD4 engagement.

CD4 binding repositions V1V2 toward the side of the trimer while rotating gp120 subunits outward and triggering changes in gp41; however, most of V1V2 and V3 are disordered in sCD4-bound cryo-EM structures (Ozorowski et al., 2017, Wang et al., 2016). Consistent with these large-scale conformational changes, the addition of sCD4 to SOSIP Envs in DEER experiments shifted the most probable distances in all spin-labeled Env motifs near the trimer apex. In motifs that are ordered in sCD4-bound structures, the most probable DEER distances were in agreement with structures; however, all distributions were broad and multimodal, indicating a high level of conformational heterogeneity in sCD4-bound complexes. Observed distances that do not correspond to sCD4-bound Env structures could represent heterogeneous, but defined, positions that were in disordered regions of cryo-EM structures of sCD4-bound Envs (Ozorowski et al., 2017, Wang et al., 2016). Heterogeneity could also be explained by increased flexibility among individual motifs and/or deviations from 3-fold symmetry in Env trimer during or after sCD4 binding. For example, in sCD4-bound complexes, short distance peaks could arise from sub-stoichiometric sCD4 binding, which could induce asymmetric Env conformations characterized by displaced motifs (e.g., V1V2 or V3) in a sCD4-bound protomer adjacent to unbound “closed” motifs in an unliganded protomer. Additional evidence supporting the existence of asymmetric Env conformations was recently provided by smFRET experiments showing that individual Env protomers on native virions adopt distinct conformations during sCD4-induced trimer opening (Ma et al., 2018).

The open sCD4-bound B41 SOSIP structure showed conformational changes in gp41 compared with closed bNAb-bound SOSIP structures (Ozorowski et al., 2017), although their magnitudes were smaller than the sCD4-induced displacements of the gp120 subunit and V1V2 region (Wang et al., 2016). The most probable distances for DEER measurements of SOSIPs with labels in gp41 in the presence of sCD4 were generally consistent with cryo-EM structures (Ozorowski et al., 2017, Wang et al., 2016). However, gp41 DEER measurements exhibited heterogeneity in the presence and absence of sCD4, with relatively minor changes upon addition of sCD4.

Comparing DEER measurements on bNAb-bound complexes with bNAb-bound Env structures (Ward and Wilson, 2017) validated and extended static structural results. DEER experiments examining the effects of the CD4bs bNAb b12 were consistent with the cryo-EM structure of a B41-b12 complex, which revealed b12 interactions with the CD4bs and V1V2 that stabilized an open Env conformation distinct from sCD4-bound complexes (Ozorowski et al., 2017). It was postulated that this conformation arises in part from b12 capturing a transient conformation (Ozorowski et al., 2017, Tran et al., 2012). However, DEER experiments showed no evidence that the V1V2, bridging sheet, or V3 regions of unliganded BG505 or B41 SOSIP Envs sampled b12-bound conformations transiently. Together with observations that BG505-173^∗^–b12 and B41-173^∗^–b12 distance distributions exhibited a peak that was not present in unliganded B41-173^∗^ distance distributions, our results suggest that the b12-bound Env conformation arises from an induced-fit mechanism rather than from capturing a transient Env conformation in equilibrium with the closed state. The emergent long-distance peaks associated with b12 binding were more populated in B41-173^∗^ than BG505-173^∗^ measurements, and addition of b12 induced greater heterogeneity in B41-306^∗^ than in BG505-306^∗^, suggesting that Env apex displacements were easier to induce in B41 than BG505.

By contrast to the b12 bNAb, addition of the CD4bs bNAb 3BNC117 or the fusion-peptide binding bNAb VRC34 had only minor effects on SOSIP conformations. However, the observation that DEER spectroscopy detected changes upon binding of VRC34 and showed that VRC34 inhibited some sCD4-induced conformational changes illustrates the sensitivity of DEER to investigate uncharacterized Env structures and effects of bNAb binding.

To address how data describing Env conformational heterogeneity relate to molecular structures, we compared results from DEER and smFRET, methods that provide distinct information related to protein structure and dynamics (Jeschke, 2012, Roy et al., 2008). DEER spectroscopy yields direct distance measurements up to ∼80Å and detects the probability of different polypeptide chain backbone conformations that reflect local, domain, and global movements in molecules in a population by measuring between small and rigid labels located in defined secondary structures (Jeschke, 2012). In contrast, smFRET is a single molecule technique that records millisecond to minute timescale changes in the relative positions of large, flexible probes separated by up to ∼80Å (Roy et al., 2008). The FRET signal is proportional to the distance between the dipole moments of the probes and can be correlated with domain and global movements; however, because the probes adopt unknown orientations relative to the polypeptide chain backbone, smFRET provides indirect distance information associated with conformational transition(s) (i.e., potential changes in FRET states might not be directly correlated with distance changes between the polypeptide chain backbones in the labeled sites). In both DEER and smFRET experiments, probe locations affect the results and interpretation of data. For example, smFRET studies of labeled virion-bound Envs described FRET changes between donor and acceptor dyes located in the V1 and V4 regions of a single protomer within each Env trimer (Figure 7A) (Munro et al., 2014) (i.e., intra-protomer changes), thus reporting on potential changes between two positions at or near the apex of an Env protomer. By contrast, DEER experiments reported here measured inter-protomer distances between three labels at six locations within soluble Env trimers, as well as an intra-protomer V1V2–V4 distance. With these differences in mind, we attempted to integrate our DEER results using SOSIP Envs with smFRET studies conducted on virion-bound Envs (Ma et al., 2018, Munro et al., 2014), combining structural data derived from crystallographic studies of closed Envs (Ward and Wilson, 2017), single-particle cryo-EM structures of sCD4-bound Envs (Ozorowski et al., 2017, Wang et al., 2016), and cryo-ET structures of both conformations on virions (Liu et al., 2008).

The smFRET studies reporting three states for the V1–V4 distance on virion Envs used labels attached to peptide linkers inserted at V1 residue 136 and V4 residue 400 (Ma et al., 2018, Munro et al., 2014), which are separated by ∼48Å in closed Env structures (measured in pdb 5CEZ; [Garces et al., 2015] between Cα atoms of residues 136 and 398; residue 400 is disordered). If changes in dye positions correlate directly with distance changes between V1 and V4, then State 1 represents the longest intra-protomer dye distance, with States 2 and 3 representing short and intermediate distances, respectively. The BG505-173^∗^+394^∗^ DEER distances were consistent with closed Env structures in which residue 173 (in V1) and residue 394 (adjacent to V4) are separated by 49Å, and assuming a closed trimer apex as seen in Env structures (Ward and Wilson, 2017) and confirmed by inter-subunit DEER measurements, 49Å is likely the smallest distance that can separate residues 173 and 394; i.e., steric constraints should prevent shorter distances separating V1 and V4. If so, then existing SOSIP (Ward and Wilson, 2017) and non-SOSIP (Lee et al., 2016) Env structures represent the smFRET state with the shortest intra-dye distance; i.e., State 2.

The observation that smFRET State 3 was populated from State 2 upon the addition of sCD4 suggests State 3 represents sCD4-bound conformation(s) (Ma et al., 2018, Munro et al., 2014), which presumably correspond to the CD4-bound open conformation observed in cryo-EM structures of sCD4-bound SOSIPs (Ozorowski et al., 2017, Wang et al., 2016) and cryo-ET structures of sCD4-bound Envs on viruses (Liu et al., 2008), which are equivalent within the ∼20Å resolution limitation of the cryo-ET-derived structures (Harris et al., 2011). The intermediate FRET associated with State 3 is consistent with (1) the increased distance between V1 and V4 in sCD4-bound Env structures resulting from V1V2 displacement from the trimer apex (Wang et al., 2016), (2) an ∼72Å V1–V4 distance measured between residues 136 and 400 in a sCD4-bound SOSIP Env structure (Wang et al., 2016) including a molecular dynamics model for the displaced V1V2 (Yokoyama et al., 2016), and (3) the appearance of long inter-subunit distances (> 70Å) in BG505-173^∗^–sCD4 and B41-173^∗^–sCD4 DEER experiments. The lower FRET signal associated with State 1 indicates an increase in the distance between probes in V1 and V4 compared with the State 3 inter-probe distance, suggesting that V1 and V4 are separated by larger distances in State 1 Envs than in CD4-bound Envs. This possibility is difficult to reconcile with the constraints of a closed trimer apex that buries the coreceptor binding site.

Intra-subunit DEER measurements between V1V2 residue 173 and V4 residue 394, which approximate the positions of smFRET dyes inserted into V1 and V4 (Figure 7A), suggested a single predominant state for the closed BG505 SOSIP apex, rather than the three states interpreted from the smFRET studies of unliganded virion-bound Envs (Munro et al., 2014). This DEER result was consistent with closed SOSIP (Ward and Wilson, 2017) and non-SOSIP (Lee et al., 2016) Env structures, as well as the closed, unliganded Env conformation on virions (Liu et al., 2008), and revealed no evidence for distinct conformational states with respect to the V1V2 – V4 distance in the absence of sCD4 as suggested by smFRET (Ma et al., 2018, Munro et al., 2014). Although this difference could relate to different V1–V4 conformational landscapes in SOSIPs compared with virion Envs, some of the discrepancy could result from size, hydrophobicity, and/or flexibility differences in DEER and smFRET labels.

Although our experiments showed no evidence of multiple states with respect to V1V2–V4 separation distances, DEER detected multiple inter-subunit distances in gp41 and neighboring regions of the gp120 inner domain of unliganded SOSIPs, suggesting conformational plasticity distal from the apex. If these results can be extended to virion-bound Envs, both smFRET State 1 and State 2 could be characterized by a closed apex, but distinguishable by differences toward the Env base. Thus movements distal to the apex might be sensed by the smFRET V4 probe, which is large and flexible enough to extend below the trimer apex and whose position could be altered by ligands that affect the stability of this region and/or by the SOSIP substitutions. Indeed, DEER experiments detected multiple states of unliganded SOSIPs in Env regions below the apex, perhaps analogous to the multiple states of virion-bound Env detected by smFRET (Ma et al., 2018, Munro et al., 2014). One way to more directly compare results from smFRET and DEER experiments would be to perform smFRET studies on SOSIPs labeled in multiple locations with smaller and less flexible dyes. Another possibility, conducting DEER experiments on spin-labeled virion Envs, is not yet feasible because the concentrations of properly folded, spin-labeled Env likely achievable in virions is insufficient for DEER measurements using currently-available technology.

In conclusion, the DEER results reported here provide previously-unavailable information, not detectable in cryo-EM and X-ray structures, which map regions of low (apex) and high (base) structural heterogeneity in SOSIP Envs currently being evaluated as immunogens. DEER measurements uncovered evidence for multiple conformations in the SOSIP Env base and demonstrated decoupling between the base and apex, indicating that SOSIP immunogens do not expose non-neutralizing apex epitopes (e.g., V3), which if extended to virion-bound Envs, would prevent membrane perturbations from triggering Env apex opening until CD4 engagement. Thus DEER measurements are informative for evaluating the conformational stability of immunogens and understanding the metastable, closed pre-fusion state of HIV-1 Env. When combined with measurements in the presence of sCD4, which revealed receptor-bound conformations in V1V2, V3, and the trimer base not seen in sCD4-bound cryo-EM structures (Ozorowski et al., 2017, Wang et al., 2016), DEER can be used to map conformational changes required for coreceptor binding and fusion between the viral and host cell membranes. Our results also demonstrated strain-specific differences in Env conformations and degrees to which bNAb and inhibitor binding alter the Env conformational landscape. This information is important for better understanding viral fusion, how we might inhibit this process to combat HIV-1 infection, and for identifying conformational differences that distinguish the most effective immunogens.

## STAR★Methods

### Key Resources Table

**Table:** 

REAGENT or RESOURCE	SOURCE	IDENTIFIER
**Chemicals, Peptides, and Recombinant Proteins**		

kifunensine	GlycoSyn	Cat#FC-034
tris(2-carboxyethyl)phosphine (TCEP)	Pierce	Cat#20491
bis(2,2,5,5-tetramethyl-3-imidazoline-1-oxyl-4-il)-disulfide	Enzo	Cat# ALX-430-102-M010
Deuterium oxide, 99.9 atom % D	Sigma-Aldrich	Cat#151882-10x0.6ML
BG505 SOSIP.664 v3.2 (A501C, T605C, I559P, R6, ΔMPER, I535M, L543N)	Bjorkman Lab	GenBank: ABA61516 and DQ208458; Sanders et al., 2013, de Taeye et al., 2015.
BG505 SOSIP.664 v3.2 (Y173C)	This paper	N/A
BG505 SOSIP.664 v3.2 (S306C)	This paper	N/A
BG505 SOSIP.664 v3.2 (T202C)	This paper	N/A
BG505 SOSIP.664 v3.2 (T106C)	This paper	N/A
BG505 SOSIP.664 v3.2 (T394C)	This paper	N/A
BG505 SOSIP.664 v3.2 (R542C)	This paper	N/A
BG505 SOSIP.664 v3.2 (E657C)	This paper	N/A
BG505 SOSIP.664 v3.2 (Y173C; T394C)		
B41 SOSIP.664 v4.2 (A501C, T605C, I559P, R6, ΔMPER, I535M, L543Q, L543N, A316W, H66R)	Bjorkman Lab	GenBank:EU576114; Pugach et al., 2015, de Taeye et al., 2015
B41 SOSIP.664 v4.2 (Y173C)	This paper	N/A
B41 SOSIP.664 v4.2 (S306C)	This paper	N/A
B41 SOSIP.664 v4.2 (T202C)	This paper	N/A
B41 SOSIP.664 v4.2 (E106C)	This paper	N/A
B41 SOSIP.664 v4.2 (R542C)	This paper	N/A
B41 SOSIP.664 v4.2 (E657C)	This paper	N/A
2G12 IgG	Bjorkman Lab	See recombinant DNA
sCD4	Bjorkman Lab	See recombinant DNA
b12 Fab	Bjorkman Lab	See recombinant DNA
3BNC117 Fab	Bjorkman Lab	See recombinant DNA
BMS-626529	APExBIO	Cat#A3253
VRC34.01 Fab	Bjorkman Lab	See recombinant DNA

**Critical Commercial Assays**		

QuikChange II Site-Directed Mutagenesis Kit	Agilent	Cat#200524

**Experimental Models: Cell Lines**		

HEK293-6E	National Research Council of Canada	License#11565
Expi293-F	ThermoFisher	Cat#A14635

**Recombinant DNA**		

pTT5 mammalian expression vector (used to express all BG505 SOSIP variants and all ligands described above)	National Research Council of Canada	N/A
pIPP4 mammalian expression vector (used to express all B41 SOSIP variants and all ligands described above)	John Moore Laboratory	Weill Cornell Medical College
2G12 IgG light chain in pTT5	Bjorkman Lab	Buchacher et al., 1994
2G12 IgG heavy chain in pTT5	Bjorkman Lab	Buchacher et al., 1994
b12 Fab light chain in pTT5	Bjorkman Lab	Burton et al., 1991
b12 Fab heavy chain in pTT5	Bjorkman Lab	Burton et al., 1991
3BNC117 Fab light chain in pTT5	Bjorkman Lab	GenBank: HE584538.1
3BNC117 Fab heavy chain in pTT5	Bjorkman Lab	GenBank: HE584537.1
VRC34.01 Fab light chain in pTT5	Bjorkman Lab	Kong et al., 2016
VRC34.01 Fab heavy chain in pTT5	Bjorkman Lab	Kong et al., 2016
sCD4 D1D2 in pTT5	Bjorkman Lab	NCBI Reference Sequence: NM_000616.4

**Software and Algorithms**		

Multiscale Modeling of Macromolecules (MMM)	Polyhach et al., 2011	http://www.epr.ethz.ch/software/mmm-older-versions.html
Pymol	Schrödinger, 2011	RRID:SCR_000305
LongDistances v.593	Christian Altenbach	http://www.biochemistry.ucla.edu/biochem/Faculty/Hubbell/
GraphPad Prism	GraphPad	RRID:SCR_002798

**Other**		

2.0/2.4 mm borosilicate capillary	Vitrocom, Mountain Lakes, NJ	Cat#S102
1.4/1.7 mm (i.d./o.d.) quartz capillary	Vitrocom, Mountain Lakes, NJ	Cat#CV1518Q
Recirculating/closed-loop helium cryocooler and compressor	Cold Edge Technologies, Allentown, PA	N/A
Elexsys 580 spectrometer	Bruker	N/A
E5106400 cavity resonator	Bruker	N/A
TWT amplifier	Applied Engineering Systems, Fort Worth, TX	N/A
Arbitrary waveform generator	Bruker	N/A
HiLoad 16/600 Superdex 200 pg column	GE Healthcare	Cat#28989335
HiTrap Q HP, 5 mL column	GE Healthcare	Cat#17115401
Bio-Spin P-6 Gel columns	Bio-Rad	Cat#7326228
Superose 6 10/300 GL column	GE Healthcare	Cat#17517201
2G12 5 ml column made in-house using using NHS-activated HP resin and 2G12 IgG	GE Healthcare	Cat#17071601
Crystal Structure of the BG505 SOSIP gp140 HIV-1 Env trimer in Complex with an early putative precursor of the PGT121 family at 3.0 Angstrom	RCSB Protein Data Bank	5CEZ
Crystal Structure of HIV-1 BG505 SOSIP.664 Prefusion Env Trimer Bound to Small Molecule HIV-1 Entry Inhibitor BMS-626529 in Complex with Human Antibodies PGT122 and 35O22 at 3.8 Angstrom	RCSB Protein Data Bank	5U7O
Crystal Structure of HIV-1 BG505 SOSIP.664 Prefusion Env Trimer Bound to Small Molecule HIV-1 Entry Inhibitor BMS-378806 in Complex with Human Antibodies PGT122 and 35O22 at 3.8 Angstrom	RCSB Protein Data Bank	5U7M
Cryo-EM model of B41 SOSIP.664 in complex with soluble CD4 (D1-D2) and fragment antigen binding variable domain of 17b	RCSB Protein Data Bank	5VN3
3.5 Angstrom Crystal Structure of a Fully and Natively Glycosylated BG505 SOSIP.664 HIV-1 Env Trimer in Complex with the Broadly Neutralizing Antibodies IOMA and 10-1074	RCSB Protein Data Bank	5T3Z
Crystal Structure of HIV-1 BG505 SOSIP.664 Prefusion Env Trimer in Complex with V3 Loop-targeting Antibody PGT122 Fab and Fusion Peptide-targeting Antibody VRC34.01 Fab	RCSB Protein Data Bank	5I8H
Crystal Structure at 3.7 A Resolution of Glycosylated HIV-1 Clade A BG505 SOSIP.664 Prefusion Env Trimer with Four Glycans (N197, N276, N362, and N462) removed in Complex with Neutralizing Antibodies 3H+109L and 35O22	RCSB Protein Data Bank	5V7J
Ectodomain of cleaved wild type JR-FL EnvdCT trimer in complex with PGT151 Fab	RCSB Protein Data Bank	5FUU
Cryo-EM structure of a BG505 Env-sCD4-17b-8ANC195 complex	RCSB Protein Data Bank	5THR
Cryo-EM model of B41 SOSIP.664 in complex with fragment antigen binding variable domain of b12	RCSB Protein Data Bank	5VN8
BG505 SOSIP.664 trimer in complex with broadly neutralizing HIV antibody 3BNC117	RCSB Protein Data Bank	5V8M
3.9 Angstrom Crystal Structure of a Fully and Natively Glycosylated BG505 SOSIP.664 HIV-1 Env Trimer in Complex with the Broadly Neutralizing Antibodies IOMA and 10-1074	RCSB Protein Data Bank	5T3X
Molecular Structure of Unliganded Native HIV-1 gp120 trimer: Spike region	Electron Microscopy Data Bank	EMD-5019
Molecular Structure of the Native HIV-1 gp120 trimer bound to CD4 and 17b: Spike region	Electron Microscopy Data Bank	EMD-5020

### Contact for Reagent and Resource Sharing

Further information and requests for resources and reagents should be directed to and will be fulfilled by Pamela J. Bjorkman (bjorkman@caltech.edu).

The Bjorkman lab cannot lawfully distribute clones in the pTT5 vector. Those wishing to obtain these clones must first obtain a license from the National Research Council of Canada (see Key Resource Table).

### Method Details

#### Protein Expression, Purification, and Spin Labeling

Genes encoding BG505 SOSIP.664 v3.2 (in vector pTT5; National Research Council of Canada) and B41 SOSIP.664 v4.2 (in vector pIPP4; John Moore Laboratory - Weill Cornell Medical College), soluble clade A and clade B gp140 trimers, respectively (de Taeye et al., 2015, Pugach et al., 2015, Sanders et al., 2013), including the ‘SOS’ substitutions (A501C_gp120_, T605C_gp41_), the ‘IP’ substitution (I559P_gp41_), the *N*-linked glycan sequence at residue 332_gp120_ (T332N_gp120_), an enhanced gp120-gp41 cleavage site (REKR to RRRRRR), and a stop codon after residue 664_gp41_ (Env numbering according to HX nomenclature), were modified to include cysteine residues at single defined positions by site-directed mutagenesis. Modified SOSIPs were expressed in transiently-transfected HEK293-6E cells (National Research Council of Canada) or Expi-293-F cells (ThermoFisher) in the presence of 5 μM kifunensine and purified by 2G12 immunoaffinity chromatography, ion exchange chromatography, and size exclusion chromatography (SEC) as previously described (Scharf et al., 2015). Introduction of cysteines did not alter SEC migration (data not shown). The protein ligands, bNAb Fabs and sCD4 domains 1 and 2, were purified from supernatants of transiently-transfected HEK293-6E cells as described (Scharf et al., 2015). The HIV-1 attachment inhibitor BMS-626529 was purchased from APExBIO.

#### SOSIP Nitroxide Spin Labeling

Purified SOSIP Envs were concentrated to ∼100 μM (gp120-gp41 protomer concentration) in Tris-buffered saline (TBS) pH 7.4 and diluted 2x in buffer containing TBS, 40 mM EDTA, and tris(2-carboxyethyl)phosphine (TCEP), resulting in a final solution with a 2x molar excess of TCEP relative to each target cysteine residue. After a 1 hour incubation at room temperature, TCEP was removed using a desalting column (Biorad) and the resulting protein solution was incubated with a 5 molar excess of bis(2,2,5,5-tetramethyl-3-imidazoline-1-oxyl-4-il)-disulfide, which yields the V1 nitroxide side chain, for 3-5 hours at room temperature and overnight at 4°C. Excess spin label was removed using Superose 6 SEC. Elution profiles of spin-labeled variants were superimposable with those recorded prior to spin labeling (data not shown). To further assess the effects of introducing cysteines into SOSIP Envs, we used a thermofluor dye-binding assay (Lavinder et al., 2009) to compare the melting temperatures of unmodified BG505 and B41 SOSIP proteins before and after TCEP reduction to the melting temperatures of their counterpart cysteine variants used for DEER experiments. The thermofluor-derived melting temperatures for cysteine-modified and TCEP-treated BG505 and B41 variants were within 2°C of the melting temperatures derived using this assay for wild-type BG505 (melting temperature = 68°C) and B41 (melting temperature = 58°C) SOSIP proteins (data not shown). Proteins were exchanged into deuterated solvent to increase the nitroxide spin-spin relaxation times, thus allowing longer times of data collection (El Mkami and Norman, 2015). Incubations of SOSIPs with bNAb, sCD4, and/or small molecule ligands were conducted for ≥ 20 hr, and samples for DEER were maintained at 4°C until being flash frozen. Deuterium-exchanged proteins and protein complexes were flash frozen in DEER capillaries within 48 hours of spin labeling to minimize dissociation of the V1 spin label.

#### Pulsed DEER Spectroscopy

For pulsed DEER spectroscopy, a 15-30 μL sample of ∼25-150 μM spin-labeled protein in a deuterated buffer solution containing 20% glycerol was placed in a 1.4/1.7 mm (i.d./o.d.) quartz capillary jacketed in a 2.0/2.4 mm borosilicate capillary (Vitrocom, Mountain Lakes, NJ) and then flash frozen in liquid nitrogen. Sample temperature was maintained at 50 K by a recirculating/closed-loop helium cryocooler and compressor system (Cold Edge Technologies, Allentown, PA). Four-pulse Q-band DEER experiments were conducted on a Bruker Elexsys 580 spectrometer fitted with a E5106400 cavity resonator. Pulse lengths were optimized via nutation experiment but ranged from 12 to 22 ns (π/2) and 24 to 44 ns (π); pulses were amplified with a TWT amplifier (Applied Engineering Systems, Fort Worth, TX). Observer frequency was set to a spectral position 2 G downfield of the low and central resonance intersection point, and the pump envelope frequency was a 50 MHz-wide square-chirp pulse (generated by a Bruker arbitrary waveform generator) set 70 MHz downfield from the observer frequency. Dipolar data were analyzed using LongDistances v.593, a custom program written by Christian Altenbach in LabVIEW (National Instruments); software available online (http://www.biochemistry.ucla.edu/biochem/Faculty/Hubbell/) and described elsewhere (Fleissner et al., 2009). Processing of dipolar evolution data (Figure S2–S6) yields distance probability distributions that reflect all interacting spins in the 15-80Å range (Jeschke, 2012). Each distribution was normalized to total area and depth of modulation (DOM), as indicated by solid lines in the distributions (Figure 2, Figure 3, Figure 4, Figure 5, Figure 6). For ease of visualization of peak distances and FWHMs, we also normalized liganded datasets to the maximum amplitude of their unliganded counterpart (dotted lines in Figure 2, Figure 3, Figure 4, Figure 5, Figure 6). Distance distributions shown in figures were made using Prism (GraphPad) and associated molecular structure figures were made using Pymol (Schrödinger, 2011).

Dipolar evolution data for mock-labeled BG505 and B41 SOSIP proteins (purified proteins containing no introduced cysteines that were subjected to the V1 labeling procedure) did not exhibit signals above background (data not shown).

#### Modeling Potential V1 Nitroxide Rotamers and Fitting Structures to EM Maps

To investigate how nitroxide side chain rotamers contributed to DEER measurements, we compared inter-subunit Cα-Cα distances (derived from measurements in Env structures from the Protein Data Bank) with inter-subunit V1 nitroxide radicals modeled into the same structures. The Multiscale Modeling of Macromolecules (MMM) program (Polyhach et al., 2011) (http://www.epr.ethz.ch/software/mmm-older-versions.html) was used to model positions of V1 rotamers at each target site. Structures of BG505 and B41 SOSIPS (pdb codes 5CEZ, 5VN8, 5VN3) were used as templates. Each target residue was mutated to cysteine using Pymol (Schrödinger, 2011). V1 spin labels were modeled onto resulting structures using MMM, generating a library of potential rotamers at each site that were used in simulations of DEER distance distributions. We used the most probable rotamer identified by MMM to measure distances between nitroxide radical atoms using Pymol (Schrödinger, 2011). For these measurements, we assumed that the probability of V1 side chains adopting a given rotamer was equivalent in all three protomers of homotrimeric Env. The analysis demonstrated consistency between Cα-Cα, V1 label - V1 label, and experimentally-determined DEER distances (Figure S1B). Thus differences in V1 rotamers are likely to contribute minimally to experimental DEER distance distributions.

To assess the similarity between atomic resolution structures and EM maps derived by cryo-ET/subtomogram averaging and to make associated figures, SOSIP Env structures (closed, pdb 5T3X (Gristick et al., 2016) and CD4-17b-bound, pdb 5VN3 (Ozorowski et al., 2017)) were fit to the EM density maps of virion-bound Envs (unliganded EMDB 5019 and CD4-17b-bound EMDB 5020 respectively) (Liu et al., 2008) using UCSF Chimera (Pettersen et al., 2004). N-glycans at Env residues 186, 137, 339, 398, 411 and 462 (disordered in 5T3X) were modeled as Man8. Coordinates and EM density for Fabs were omitted for clarity.

## Supplemental Information

Document S1. Figures S1–S6

Document S2. Article plus Supplemental Information
