# Supplementary material for: DEER Spectroscopy Measurements Reveal Multiple Conformations of HIV-1 SOSIP Envelopes that Show Similarities with Envelopes on Native Virions
Source: Immunity. 2018 Aug 21;49(2):235–246.e4. doi: 10.1016/j.immuni.2018.06.017 (PMC6104740; doi:10.1016/j.immuni.2018.06.017)
Supplement: Document S1. Figures S1–S6 [file mmc1.pdf]

Immunity, Volume *49*

Supplemental Information

DEER Spectroscopy Measurements Reveal Multiple Conformations of HIV-1 SOSIP Envelopes that Show Similarities with Envelopes on Native Virions

Beth M. Stadtmueller, Michael D. Bridges, Kim-Marie Dam, Michael T. Lerch, Kathryn E. Huey-Tubman, Wayne L. Hubbell, and Pamela J. Bjorkman

SUPPLEMENTAL FIGURE LEGENDS

Supplemental Figure 1, related to Figure 1. Chemical structures of labels used for DEER and smFRET. (A) The V1 nitroxide side chain attached to a polypeptide backbone cysteine residue. (B) Top: Measurements of target sites from a closed BG505 structure (pdb code 5CEZ), in which the V1 nitroxide side chain was computationally attached using MMM (Polyhach et al., 2011). The measurements between $C\alpha$ atoms (red lines) and nitroxide radicals (blue lines) are indicated for each target site. Bottom: Table summarizing the number of V1 rotamers identified by MMM, the most probable distance for each site predicted in a DEER simulation, and the radical-radical atom distance between each site for the most probable V1 rotamer, the $C\alpha$-$C\alpha$ distance between each site measured in pdb 5CEZ and the average distance and standard deviation measured in seven pdbs (5CEZ, 5T3Z, 5I8H, 5V7J, 5FUU, 5U7M, 5U7O). All predictions agree within 2-3 Å with measured backbone $C\alpha$ distances except for site 202 in which case the BG505-202* DEER data were consistent with the $C\alpha$-$C\alpha$ distance (Figure 1, 2). (C) Donor and acceptor smFRET probes attached to a Gln within a peptide linker inserted at residue 136 in V1 (top) and a Ser within a peptide linker inserted at residue 400 in V4 (bottom) in virion-bound Envs (Munro et al., 2014). The COT (1,3,5,7 cyclooctatetraene) moiety adds hydrophobicity to the V4 probe.

Supplementary Figure 2, related to Figure 2. Background corrected dipolar evolution data for SOSIP Envs and SOSIP Envs incubated with sCD4. Background corrected dipolar evolution data (black traces) for each indicated sample with fits to data shown in red. An important feature of the dipolar evolution function is the depth of modulation (DOM). For given instrumental settings, the DOM is proportional to the number of interacting spin pairs that lie within the detectable distance limit of the DEER experiment. The vertical axis of each plot in the figure is scaled to show details and the quality of fit; the DOM is the full amplitude of change on

the vertical axis. The maximum DOM for a quantitatively labeled trimer was ~80% for the instrument and settings used in these experiments. Distance distributions obtained from analysis of low DOM data (<10%) are implicitly less reliable. Low DOM can be attributed to a small equilibrium population within the detectable distance limit or can indicate low spin labeling efficiency. In situations in which the addition of a ligand to a labeled Env causes a dramatic drop in DOM depth (e.g., sCD4 added to BG505-173* or B41-173*), the DOM drop strongly suggests that the majority of spin pairs moved out of the detectable range in the presence of ligand. Data with large DOM can be problematic in tri-radical systems such as the labeled Env trimers studied here because, for a rigid and completely-labeled system of three interacting spins, in addition to a single major peak in the distribution observed at a distance corresponding to the legs of the equilateral triangle representing the three labels, a smaller "ghost peak" will also appear at a distance ~80% of the real peak (Jeschke, 2012). The ghost peak amplitude becomes insignificant for trimers that have low labeling efficiency and thus low DOM (<~0.50), but the major peak remains unchanged in position. Thus, under-labeling of the sample simplifies the analysis. The labeling efficiency high enough to observe ghost peaks in only two cases reported here: B41-306* (Figure 2E, ~31 Å) and B41-306* + b12 (Figure 3F, broad shoulder to peak ~20-30 Å).

Supplemental Figure 3, related to Figure 3. Background corrected dipolar evolution data for SOSIP Env incubated with CD4bs bNAbs. Dipolar evolution data (black traces) for all trimer measurements shown in Figure 3 with fits to data shown in red. A potential ghost peak is observed in panel F between 20-30 Å. See Supplemental Figure 2 legend for discussion of DOM and ghost peaks.

Supplemental Figure 4, related to Figure 4. Background corrected dipolar evolution data for SOSIP Env incubated with the small molecule inhibitor BMS-626529. Dipolar evolution

data (black traces) for all trimer measurements shown in Figure 4 with fits to data shown in red. See Supplemental Figure 2 legend for a discussion of DOM.

Supplemental Figure 5, related to Figure 5. Background corrected dipolar evolution data for SOSIP Env incubated with VRC34 and/or sCD4. Dipolar evolution data (black traces) for all trimer measurements shown in Figure 5 with fits to data shown in red. See Supplemental Figure 2 legend for a discussion of DOM.

Supplemental Figure 6, related to Figure 6. Background corrected dipolar evolution data for BG505-173*+394*, Dipolar evolution data (black traces) for all trimer measurements shown in Figure 6 with fits to data shown in red. See Supplemental Figure 2 legend for a discussion of DOM.

Figure S1

A

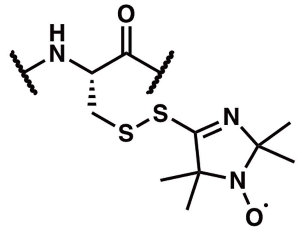

B

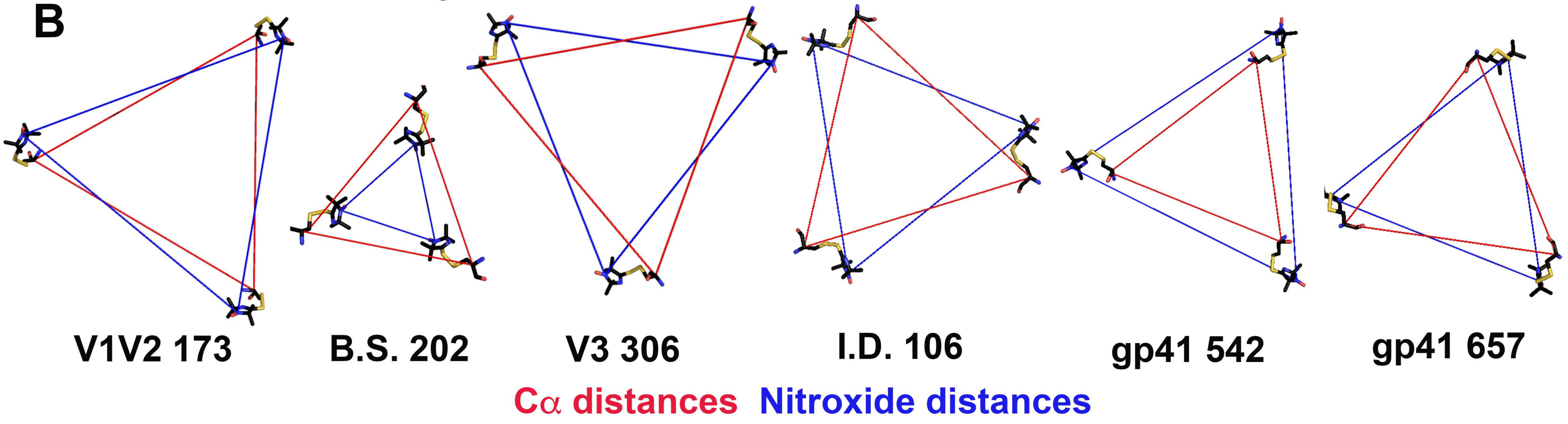

Target Site	MMM # Predicted V1 Nitroxide Rotamers	MMM Predicted DEER Distance (Å)	Nitroxide Distance (Å) (most probable rotamer)	Cα- Cα Distance (Å) (pdb: 5CEZ)	Cα- Cα Distance (Å) (pdbs: 5CEZ, 5T3Z, 5I8H, 5V7J, 5FUU, 5U7M, 5U7O)
V1V2 173	3	36	37	36	36 ± 1.1
B.S. 202	1	12	13	21	21 ± 0.6
V3 306	16	37	36	36	37 ± 0.6
I.D. 106	10	30	29	29	30 ± 0.2
gp41 542	13	34	36	25	25 ± 0.8
gp41 657	17	31	30	27	26 ± 2.8

C

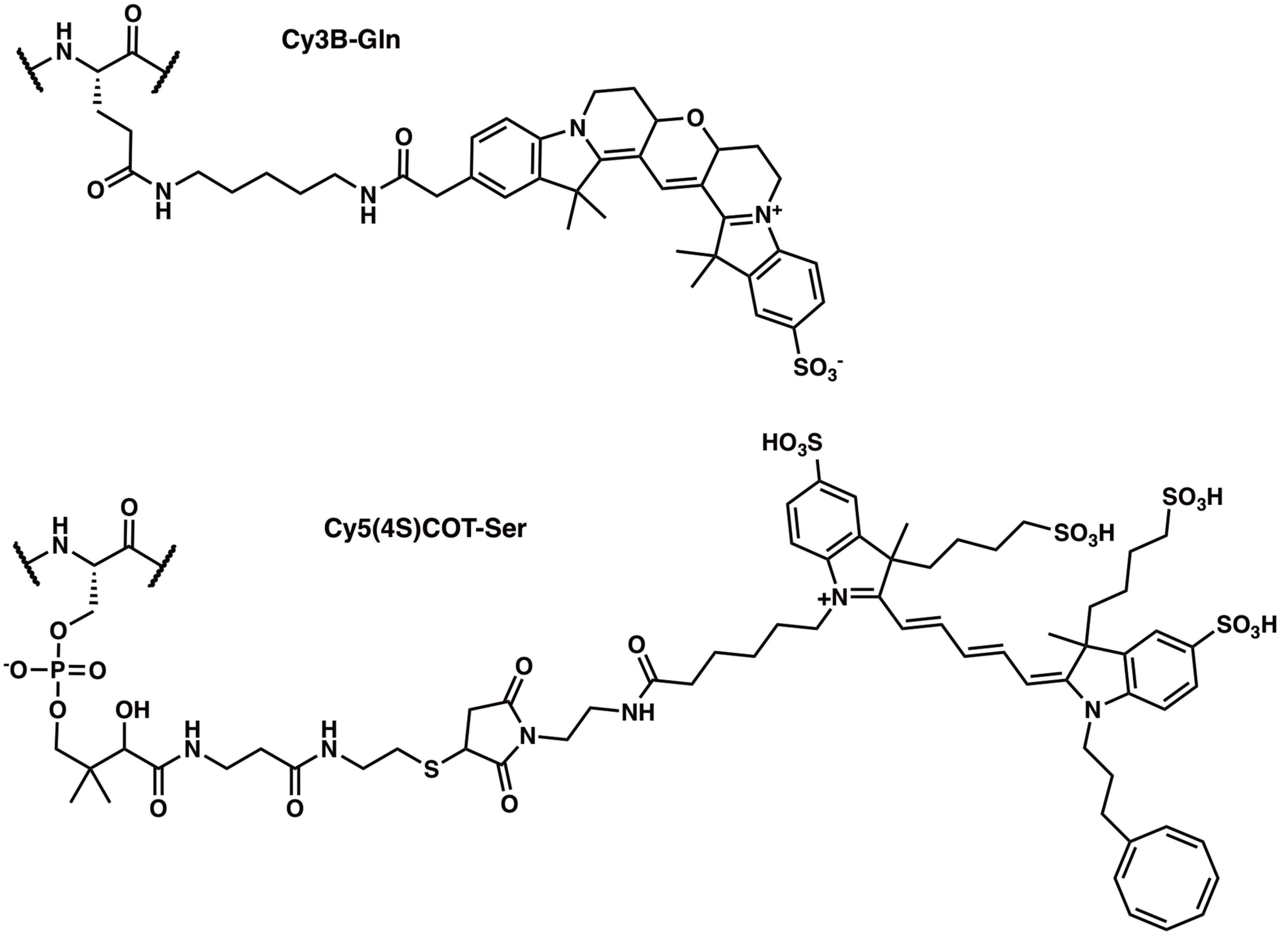

Figure S2

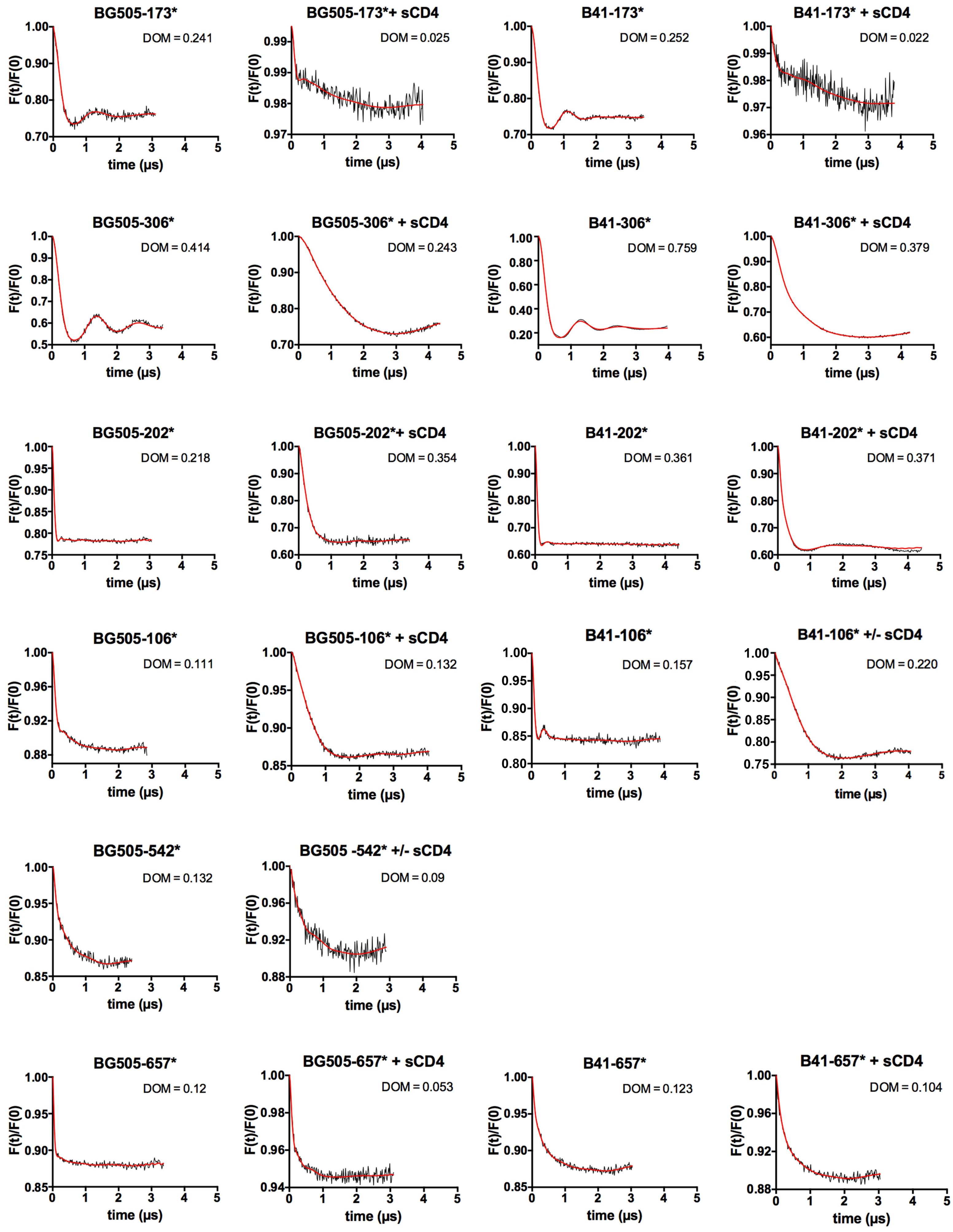

BG505-173*
DOM = 0.241
BG505-173*+ sCD4
DOM = 0.025
B41-173*
DOM = 0.252
B41-173* + sCD4
DOM = 0.022
BG505-306*
DOM = 0.414
BG505-306* + sCD4
DOM = 0.243
B41-306*
DOM = 0.759
B41-306* + sCD4
DOM = 0.379
BG505-202*
DOM = 0.218
BG505-202*+ sCD4
DOM = 0.354
B41-202*
DOM = 0.361
B41-202* + sCD4
DOM = 0.371
BG505-106*
DOM = 0.111
BG505-106* + sCD4
DOM = 0.132
B41-106*
DOM = 0.157
B41-106* +/- sCD4
DOM = 0.220
BG505-542*
DOM = 0.132
BG505 -542* +/- sCD4
DOM = 0.09
BG505-657*
DOM = 0.12
BG505-657* + sCD4
DOM = 0.053
B41-657*
DOM = 0.123
B41-657* + sCD4
DOM = 0.104
F(t)/F(0)
time (µs)

Figure S3

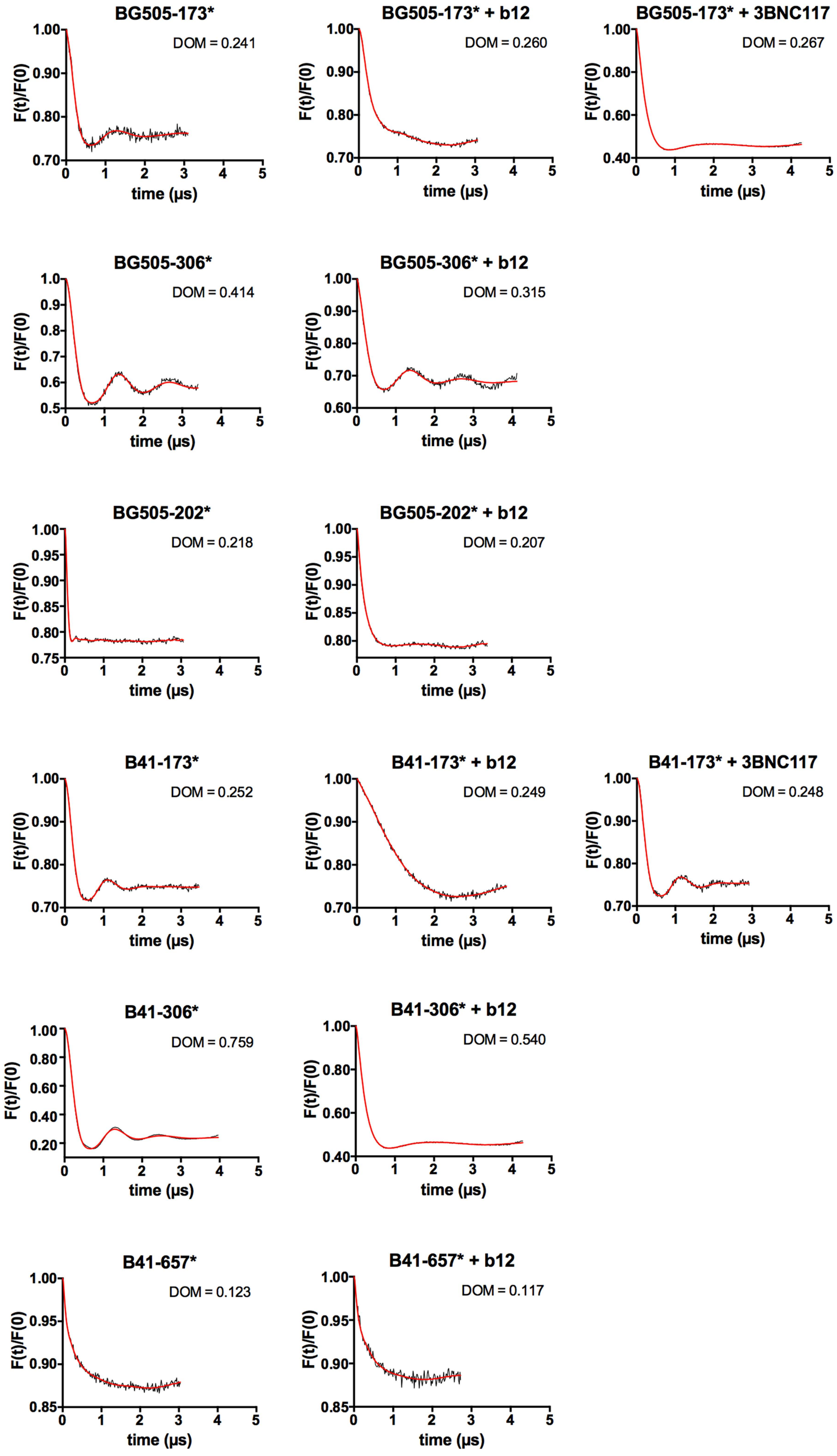

BG505-173*
DOM = 0.241
BG505-173* + b12
DOM = 0.260
BG505-173* + 3BNC117
DOM = 0.267
BG505-306*
DOM = 0.414
BG505-306* + b12
DOM = 0.315
BG505-202*
DOM = 0.218
BG505-202* + b12
DOM = 0.207
B41-173*
DOM = 0.252
B41-173* + b12
DOM = 0.249
B41-173* + 3BNC117
DOM = 0.248
B41-306*
DOM = 0.759
B41-306* + b12
DOM = 0.540
B41-657*
DOM = 0.123
B41-657* + b12
DOM = 0.117
F(t)/F(0)
time (μs)

Figure S4

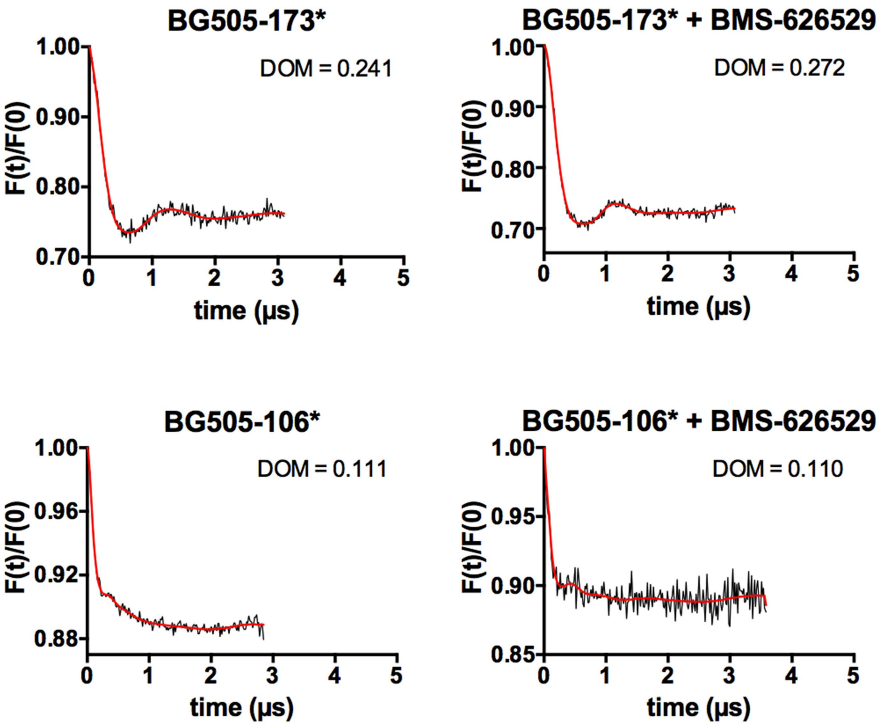

BG505-173*
DOM = 0.241
F(t)/F(0)
1.00
0.90
0.80
0.70
0
1
2
3
4
5
time (µs)
BG505-173* + BMS-626529
DOM = 0.272
F(t)/F(0)
1.00
0.90
0.80
0.70
0
1
2
3
4
5
time (µs)
BG505-106*
DOM = 0.111
F(t)/F(0)
1.00
0.96
0.92
0.88
0
1
2
3
4
5
time (µs)
BG505-106* + BMS-626529
DOM = 0.110
F(t)/F(0)
1.00
0.95
0.90
0.85
0
1
2
3
4
5
time (µs)

Figure S5

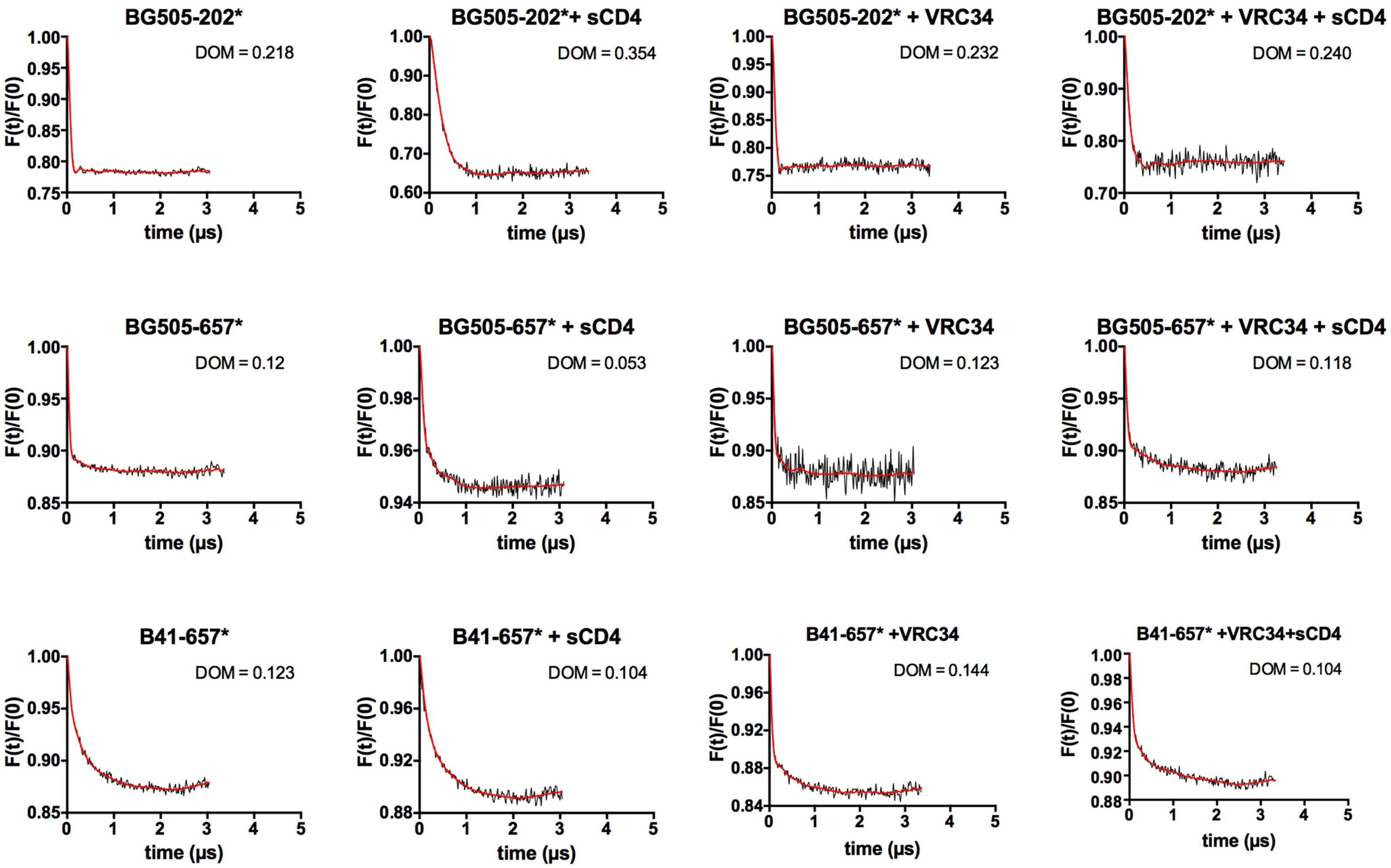

BG505-202*
DOM = 0.218
BG505-202*+ sCD4
DOM = 0.354
BG505-202* + VRC34
DOM = 0.232
BG505-202* + VRC34 + sCD4
DOM = 0.240
BG505-657*
DOM = 0.12
BG505-657* + sCD4
DOM = 0.053
BG505-657* + VRC34
DOM = 0.123
BG505-657* + VRC34 + sCD4
DOM = 0.118
B41-657*
DOM = 0.123
B41-657* + sCD4
DOM = 0.104
B41-657* +VRC34
DOM = 0.144
B41-657* +VRC34+sCD4
DOM = 0.104
F(t)/F(0)
time (μs)

Figure S6

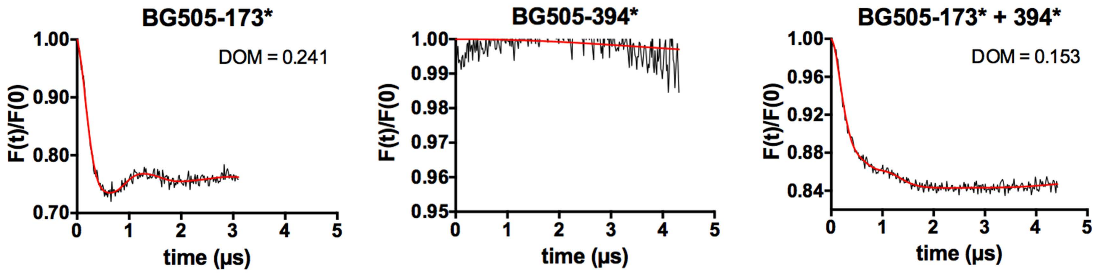

BG505-173*
DOM = 0.241
F(t)/F(0)
1.00
0.90
0.80
0.70
0
1
2
3
4
5
time (μs)
BG505-394*
F(t)/F(0)
1.00
0.99
0.98
0.97
0.96
0.95
0
1
2
3
4
5
time (μs)
BG505-173* + 394*
DOM = 0.153
F(t)/F(0)
1.00
0.96
0.92
0.88
0.84
0
1
2
3
4
5
time (μs)
